# PFOS and PFOA exposure induces liver injury and sex-dependent immune effects in C57BL/6 mice

**DOI:** 10.1016/j.isci.2026.114693

**Published:** 2026-01-14

**Authors:** Amélie Blais, Allison Loan, Eunnara Cho, Asia Woodtke, Houman Moteshareie, Lauren M. Bradford, Gong Zhang, Guillaume Pelletier, Martha Navarro, Matthew J. Meier, Andy Nong, Rocio Aranda-Rodriguez, Kristin M. Eccles, David Prescott, Azam F. Tayabali

**Affiliations:** 1 Environmental Health Science and Research Bureau, Healthy Environments and Consumer Safety Branch, Health Canada, Ottawa, ON, Canada; 2 Department of Electrical and Computer Engineering, Université de Sherbrooke, Sherbrooke, QC, Canada; 3 Bureau of Chemical Safety, Health Products and Food Branch, Health Canada, Ottawa, ON, Canada

**Keywords:** environmental toxicology, molecular toxicology, toxic injury

## Abstract

Per- and polyfluoroalkyl substances (PFASs) are persistent environmental contaminants. Perfluorooctanoic acid (PFOA) and perfluorooctanesulfonic acid (PFOS), representing carboxylate and sulfonate subclasses, are among the most frequently detected PFASs in biomonitoring studies. We characterized the hepatotoxicity and immunotoxicity of PFOS and PFOA in male and female C57BL/6-Elite mice exposed via oral gavage for 28 (1.5 mg/kg/day) or 56 days (0.166–1.5 mg/kg/day). Both compounds caused pancreatic atrophy, hepatomegaly, elevated serum biomarkers of liver injury, and decreased serum triglyceride levels across sexes and exposure durations. Liver transcriptomics revealed enrichment of PPAR signaling, lipid metabolism disruption, and AGE-RAGE pathways. Immunotoxicity assessments showed PFOS-induced cytokine suppression (interleukin-4 [IL-4], IL-17α, tumor necrosis factor-alpha [TNF-α], and monocyte chemoattractant protein-1 [MCP-1]) in males, while females exhibited minimal cytokine changes but altered thymocyte development. Overall, PFASs caused sex-independent hepatic and pancreatic toxicity but sex-dependent immune effects. Limitations include asymmetric dosing and lack of estrous monitoring; future studies should integrate histopathology and gene expression confirmatory analysis.

## Highlights

• PFOA and PFOS show distinct toxicokinetics, despite equivalent dosing
• Both PFASs induced hepatomegaly and disrupted ALP, ALT, and TGL
• PFOS and PFOA exposure caused pancreatic atrophy
• Immune effects may be sex-specific: males altered cytokines, females altered thymocytes

## Graphical abstract

**Figure:**
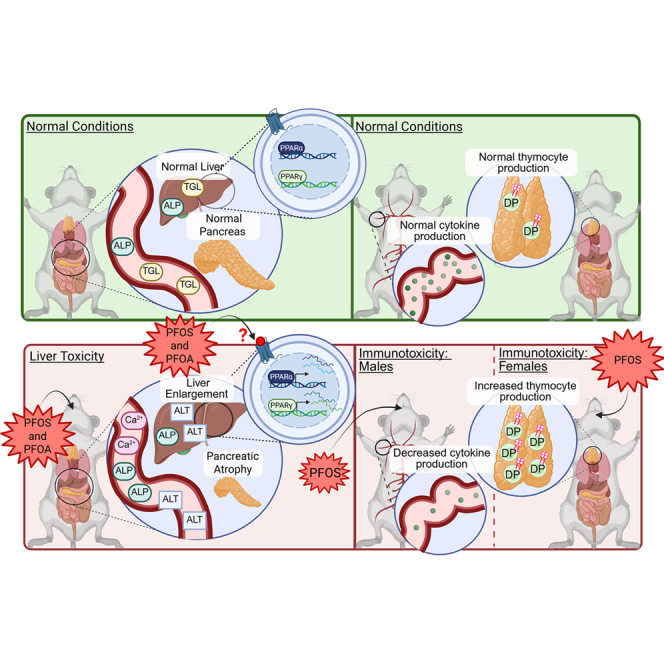

## Introduction

Per- and poly-fluorinated alkyl substances (PFASs) are a class of human-made chemicals recognized for their extreme chemical and thermal stability and their ability to repel both water and oil, making them an ideal constituent of lubricants, surfactants, firefighting foams, stain-resistant items, and non-stick coatings. However, these same properties also contribute to their persistence as contaminants in the environment and within living organisms, leading to their designation as “forever chemicals” in scientific and popular literature. Although largely phased out of production, perfluorooctane sulfonic acid (PFOS) and perfluorooctanoic acid (PFOA) remain the most abundant PFAS detected in human biomonitoring studies, including the National Health and Nutrition Environment Survey (NHANES) and the Canada Health Measures Survey (CHMS) PFAS.^1^^,^^2^^,^^3^

Given the persistence and ubiquity of PFASs, considerable attention has been given to their health effects. Epidemiological studies have associated PFAS exposure with a wide range of adverse outcomes, including chronic kidney disease, preeclampsia, thyroid dysfunction, metabolic disorders, obesity, and cancer.^4^^,^^5^ Moreover, recent studies have demonstrated PFAS-induced pancreatic toxicity, including decreased size, reduced insulin secretion, tumor production, and pancreatic cancer.^6^^,^^7^^,^^8^ Among the primary biological targets of PFAS exposure are the immune and hepatic systems.^3^ While immune-related effects are often among the most sensitive endpoints in humans, liver toxicity frequently appears as an early and prominent effect in rodent models exposed to PFASs.^9^^,^^10^

A commonly studied immune endpoint is the suppressed T cell-dependent antibody response (TDAR), which results in decreased levels of immunoglobulin M (IgM). This suppression occurs following exposure to PFOS^11^^,^^12^^,^^13^^,^^14^ and PFOA.^15^^,^^16^ However, some studies indicate that there is no effect at lower doses, suggesting dose-dependent immunosuppression.^17^ In addition to antibody suppression, PFASs have been associated with perturbed cytokine production^18^^,^^19^^,^^20^^,^^21^ and reduced weight of primary immune organs, including the thymus and the spleen.^22^ Several underlying mechanisms have been proposed to explain these phenotypic changes, namely, modulation of nuclear factor κB (NF-κB) and proliferator-activated receptors (PPARs) signaling, changes in calcium (Ca^2+^) signaling, and fatty acid metabolism.^23^ Overall, these findings on PFAS-induced immunotoxicity suggest that PFASs have broad immunomodulatory effects.

The liver, a peripheral immune organ, is a primary site of PFAS accumulation and toxicity. Numerous rodent and human studies report PFAS-induced hepatic injury, often marked by hepatomegaly and elevated serum liver enzymes such as alanine aminotransferase (ALT) and alkaline phosphatase (ALP).^24^^,^^25^^,^^26^^,^^27^^,^^28^^,^^29^^,^^30^^,^^31^ Several underlying mechanisms including lipid accumulation, oxidative stress, and nuclear receptor activation, such as pregnane X receptor (PXR) activation, have been proposed.^32^^,^^33^ While hepatic effects of PFASs are well-documented, sex-specific differences in these outcomes remain poorly characterized.

Sex-based differences in immune function and liver physiology are well-established, yet many PFAS toxicology studies have not been designed to detect sex-specific responses. Key TDAR studies demonstrating reduced antibody production in response to PFOS have been conducted primarily in male mice, while PFOA studies have typically used either male^16^ or female mice,^15^^,^^18^^,^^34^^,^^35^ and less commonly, both. However, in humans, PFAS levels differ between males and females, with males typically showing higher blood concentrations despite there being no appreciable differences in PFAS metabolism between sexes.^36^ Moreover, epidemiological data show that there are differences in the adverse effects observed between the two sexes.^37^ Thus, additional research is needed to evaluate how sex may influence immune outcomes following exposure to different PFAS. In addition to sex, the duration of PFAS exposure is another critical factor that alters toxicological outcomes but remains understudied. Immune and hepatic effects may evolve over time, with specific outcomes only becoming evident after a sub-chronic exposure duration.

To address these two gaps in exposure duration and sex-specific outcomes, we aimed to investigate both short-term (28 days) and sub-chronic (56 days) effect of PFOS and PFOA exposure in male and female mice, focusing on hepatic function at both time points and immune function primarily at 56 days. This study offers insight into potential sex- and time-specific vulnerabilities, supporting more refined risk assessments for these persistent environmental contaminants.

## Results

**Figure 1:**
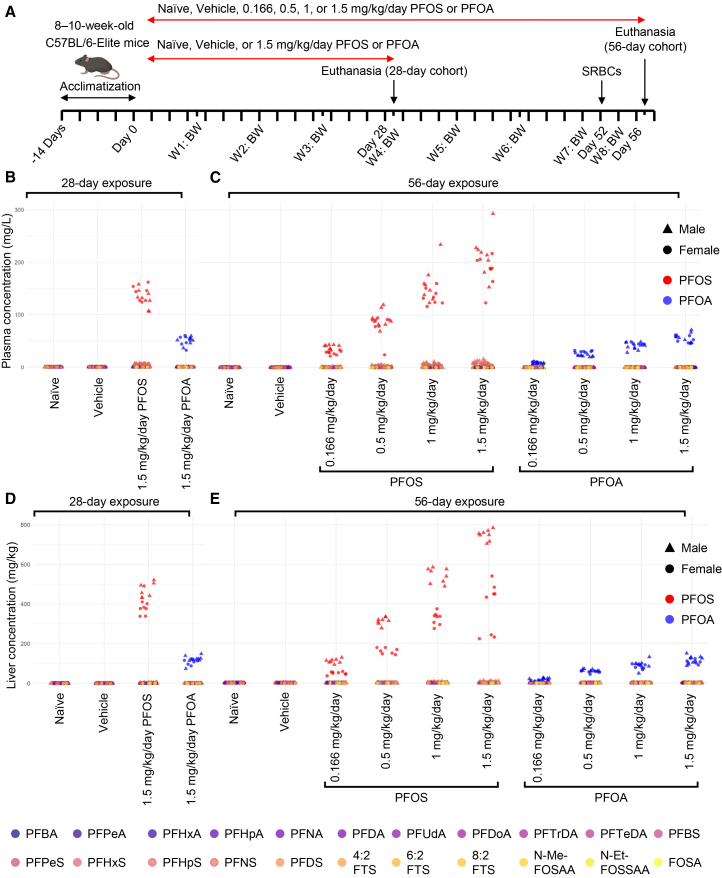
PFOA and PFOS concentrations in plasma and liver following exposure (A) Schematic of experimental timeline created in BioRender. Loan, A. (2026) https://BioRender.com/141xzxf. (B and C) PFAS concentrations in the plasma of mice treated for 28 (B) or 56 (C) days with PFOA or PFOS. *n* = 15–16 mice/group. (D and E) PFAS concentrations in the livers of mice treated for 28 (D) or 56 (E) days with PFOA or PFOS. Colored by PFAS. *n* = 15–16 mice/group. BW, body weight; FOSA, perfluorooctanesulfonamide; N-EtFOSAA, N-ethyl perfluorooctanesulfonamidoacetic acid; N-MeFOSAA, N-methyl perfluorooctanesulfonamidoacetic acid; PFBA, perfluorobutanoic acid; PFDA, perfluorodecanoic acid; PFDS, perfluorodecanesulfonic acid; PFDoA, perfluorododecanoic acid; PFHpA, perfluoroheptanoic acid; PFHpS, perfluoroheptanesulfonic acid; PFHxA, perfluorohexanoic acid; PFHxS, perfluorohexanesulfonic acid; PFNA, perfluorononanoic acid; PFNS, perfluorononanesulfonic acid; PFOA, perfluorooctanoic acid; PFOS, perfluorooctanesulfonic acid; PFPeA, perfluoropentanoic acid; PFPeS, perfluoropentanesulfonic acid; PFTeDA, perfluorotetradecanoic acid; PFTrDA, perfluorotridecanoic acid; PFUdA, perfluoroundecanoic acid; SHRBc, sheep red blood cells; 4:2 FTS, 4:2 fluorotelomer sulfonate; 6:2 FTS, 6:2 fluorotelomer sulfonate; 8:2 FTS, 8:2 fluorotelomer sulfonate.

The effects of exposure to PFOA and PFOS were assessed over both a short-term (28 days) and sub-chronic (56 days) period. Experimental mice were given oral doses of 1.5 mg/kg/day PFOA or PFOS for 28 days or 0.166, 0.5, 1.0, or 1.5 mg/kg/day of PFOA or PFOS for 56 days (Figure 1A). To assess tolerance to PFAS exposure, body weight and water consumption were monitored throughout the study. Relative to the vehicle, no changes in median body weight were observed in either male or female mice following 28-day (Figure S1A) or 56-day (Figure S1B) exposure to PFOA or PFOS. Similarly, relative to the vehicle, water consumption remained unchanged at both time points (Figures S1C and S1D).

To confirm appropriate PFAS exposure, liquid chromatography-tandem mass spectrometry (LC-MS/MS) analysis was performed on plasma and liver samples. Analysis of 24 PFAS compounds confirmed elevated levels of PFOA and PFOS in the plasma (Figures 1B and 1C) and liver (Figures 1D and 1E) of their respective treatment groups, while concentrations of other background PFAS remained minimal (Table S2). At the 56-day time point, average plasma concentrations of PFOS reached up to 199.69 mg/L in the PFOS 1.5 mg/kg/day group, while PFOA peaked at 55.27 mg/L in the PFOA 1.5 mg/kg/day group. Corresponding liver concentrations were markedly higher for PFOS, with levels up to 565.59 mg/kg, while PFOA reached a maximum of 113.43 mg/kg in the same group. Background levels in naive and vehicle controls remained low in both matrices, with plasma PFOS and PFOA below 0.01 mg/L, and liver concentrations below 0.00001 mg/kg. Notably, in the 56-day treatment cohort, both PFOS and PFOA exhibited strong dose-dependent increases in concentrations across all treatment groups, with plasma and liver levels rising steadily from the lowest (0.166 mg/kg/day) to the highest (1.5 mg/kg/day) administered doses (Figures 1B–1E). Despite being administered at the same concentration, PFOA accumulates to a much lower extent than PFOS in the liver and plasma, likely due to its faster elimination and lower affinity for plasma proteins compared to PFOS.^38^^,^^39^^,^^40^

### PFOA- and PFOS-induced hepatotoxicity

**Figure 2:**
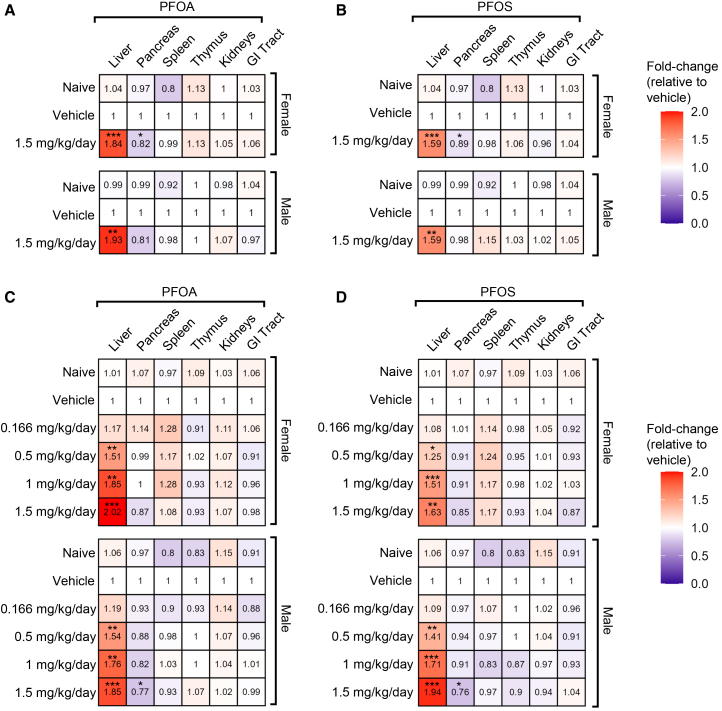
PFOA and PFOS exposure results in hepatomegaly and pancreatic atrophy Median-fold change (vs. same-sex vehicle) of liver, pancreas, spleen, thymus, kidneys, and GI tract normalized to euthanasia body weight in PFOA (A and C) and PFOS (B and D) exposed mice following 28 (A and B) or 56 (C and D) days of exposure. *n* = 8 mice/group. Statistical significance was assessed using the Kruskal-Wallis test with Dunn’s multiple comparisons (control: vehicle). Data are presented as median-fold change. ∗*p* ≤ 0.05; ∗∗*p* ≤ 0.01; ∗∗∗*p* ≤ 0.001. GI, gastrointestinal tract; PFOA, perfluorooctanoic acid; PFOS, perfluorooctanesulfonic acid.

To broadly characterize the effect of PFOA and PFOS on target organs, the weight of the liver, pancreas, spleen, thymus, kidneys, and gastrointestinal (GI) tract was recorded at euthanasia. Although there was no change in overall body weight, exposure to PFOA and PFOS led to an increase in the liver-to-body weight ratio in both male and female mice after 28 days (Figures 2A, 2B, S2A, and S2B) and a dose-dependent increase after 56 days (Figures 2C, 2D, S2C, and S2D). After 56 days of exposure, a statistically significant enlargement of the liver was observed, beginning at a dose of 0.5 mg/kg/day for both PFOA (Figures 2C and S2C) and PFOS (Figures 2D and S2D).

A decrease in the pancreas-to-body weight ratio was observed in female mice after 28 days of treatment (Figures 2A, 2B, S2A, and S2B). After 56 days, both male and female mice exhibited a downward trend in pancreas weight at a dosage of 1.5 mg/kg/day, although statistical significance was only observed in males (Figures 2C, 2D, S2C, and S2D). Additionally, no significant changes were observed in the weight of the spleen, thymus, kidneys, or GI tract at either time point (Figures 2A–2D, S2A, and S2D).

Following the observed increase in liver weight after 28 and 56 days of exposure, a panel of serum biomarkers was measured to evaluate liver toxicity. These included ALP, ALT, Ca^2+^, cholesterol (CHOL), free thyroxine (FT4), lactate dehydrogenase (LDH), total bilirubin (TBI), and triglycerides (TGL).

**Figure 3:**
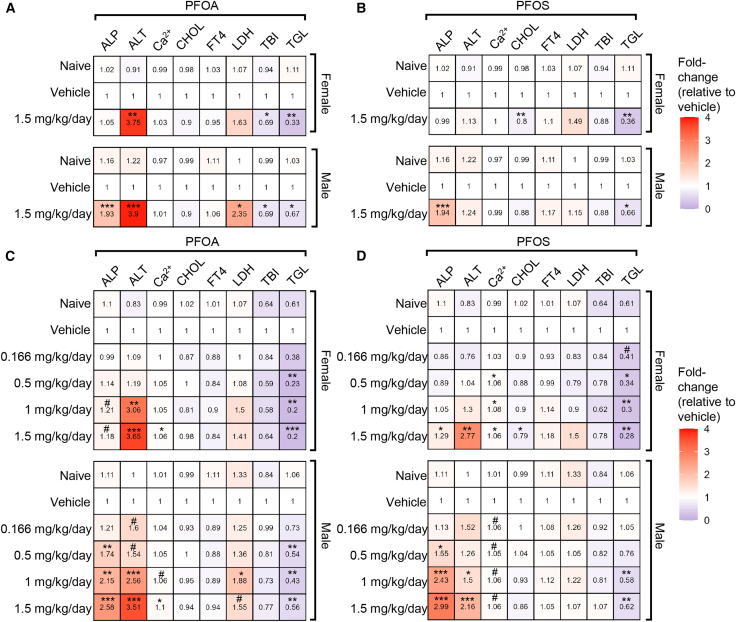
PFOA and PFOS-induced liver toxicity Median-fold change (vs. same-sex vehicle) of serum ALP, ALT, Ca^2+^, CHOL, FT4, LDH, TBI, and TGL at euthanasia in PFOA (A and C) and PFOS (B and D) exposed mice following 28 (A and B) or 56 (C and D) days of exposure. *n* = 8 mice/group. Statistical significance was assessed using the Kruskal-Wallis test with Dunn’s multiple comparisons (control: vehicle). Data are presented as median-fold change. #*p* ≤ 0.099; ∗*p* ≤ 0.05; ∗∗*p* ≤ 0.01; ∗∗∗*p* ≤ 0.001. ALP, alkaline phosphatase; ALT, alanine aminotransferase; Ca^2+^, calcium; CHOL, cholesterol; FT4, free thyroxine; LDH, lactate dehydrogenase; PFOA, perfluorooctanoic acid; PFOS, perfluorooctanesulfonic acid; TBI, total bilirubin; TGL, triglycerides.

In the 28-day treatment cohort, ALP, a marker of hepatobiliary function, was significantly elevated in male mice following exposure to both PFOA and PFOS at a dose of 1.5 mg/kg/day (Figures 3A and 3B). ALT levels, which indicate hepatocellular injury, increased in both sexes after PFOA exposure. In contrast, TBI levels were significantly decreased in PFOA-treated mice, while they remained unchanged after PFOS exposure. TGL levels declined across all treatment groups. No significant changes were observed in serum Ca^2+^ or FT4 levels in either sex under any exposure condition (Figures 3A, 3B, S3A, and S3B).

In the 56-day cohort, ALP levels increased in a dose-dependent manner in male mice exposed to either PFOA or PFOS, with significant elevations observed at a dose of 0.5 mg/kg/day. In female mice, this increase was significant at 1.5 mg/kg/day PFOS, while ALP levels in PFOA-exposed females had only a modest increase at a 95% confidence interval (CI; *p* = 0.09). ALT levels were significantly elevated in both sexes starting at a dose of 1 mg/kg/day across most treatment conditions. Serum Ca^2+^ levels were significantly increased at the highest doses of both chemicals in both sexes, except in PFOS-exposed males, who showed a non-statistically significant upward trend at α = 0.05 (*p* = 0.07). TGL levels decreased in a dose-dependent manner in both sexes, beginning at a dose of 0.5 mg/kg/day for PFOA and 1 mg/kg/day for PFOS. No significant changes were observed in FT4, LDH, or TBI levels in either sex under any exposure condition (Figures 3C, 3D, S4A, and S4B).

To determine whether differences in internal PFAS concentrations modulate key liver injury marker responses, benchmark dose (BMD) values were plotted for ALP and ALT, calculated using both nominal exposure concentrations and measured liver PFAS levels. For PFOA, BMDs derived from liver concentrations were 53.95 mg/kg/day for ALP and 62.82 mg/kg/day for ALT. In contrast, BMDs for PFOS were substantially higher, at 361.01 mg/kg/day for ALP and 429.51 mg/kg/day for ALT (Figure S4C). These results highlight the substantial differences between chemicals in the tissue dose required to elicit equivalent biomarker responses, indicating that observed differences at nominal doses are not solely attributable to bioavailability.

To further examine how these changes relate to underlying molecular mechanisms, transcriptomic profiling was conducted on the livers of experimental mice treated with 1.5 mg/kg/day PFOA or PFOS for 28 days, or with 0.166, 0.5, 1.0, or 1.5 mg/kg/day of PFOA or PFOS for 56 days (Figures S5A and S5B). Differentially expressed genes (DEGs) were discriminated by a linear fold-change (FC) > 1.5 and an adjusted *p* value (*p*_adj_) < 0.05.

**Figure 4:**
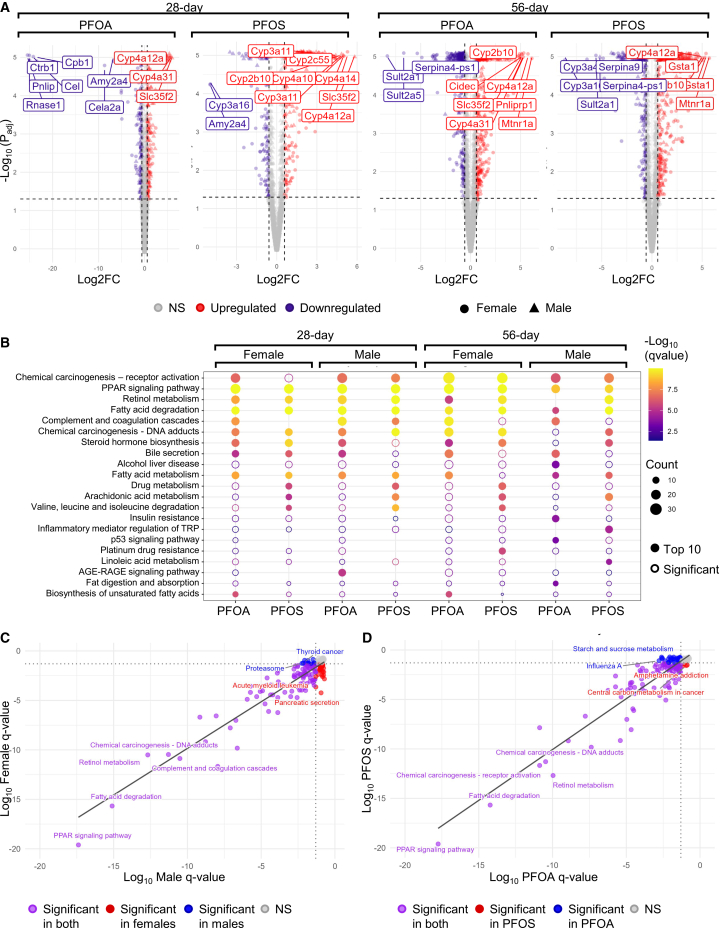
Transcriptomic analysis of PFAS-exposed mouse livers (A) Volcano plots showing differentially expressed genes (DEGs) between vehicle control (0 mg/kg/day) and 1.5 mg/kg/day PFOA or PFOS after 28 or 56 days of exposure. DEGs were defined as those with an FC > 1.5 and adjusted *p*_adj_ < 0.05. (B) Dot plots showing the top 10 significantly enriched Kyoto Encyclopedia of Genes and Genomes (KEGG) pathways (*q* value <0.05) for each condition, based on both upregulated and downregulated DEGs. Dot size reflects the number of DEGs in the pathway; color indicates the *q* value from enrichment analysis. Open circles denote significantly enriched pathways that fall outside the top 10. (C and D) Scatter plots illustrating Log_10_-transformed minimum FDR-adjusted *q* values by (C) sex and (D) PFAS type. The dotted lines indicate the standard FDR significance threshold (*q* value = 0.05), and labels indicate the top significant pathways discriminated by *q* value. FC, fold change; *p*_adj_, adjusted *p* value; PFASs, per- and polyfluoroalkyl substances; PFOA, perfluorooctanoic acid; PFOS, perfluorooctanesulfonic acid.

In the 28-day cohort, comparison between the vehicle and high-dose groups identified 299 and 188 DEGs in PFOA- and PFOS-treated females, respectively, and 374 and 214 DEGs in PFOA- and PFOS-treated males (Figures 4A, S5C, and S5D). In the 56-day cohort, a dose-dependent increase in DEGs was observed for both PFAS compounds tested. At the highest dose (1.5 mg/kg/day), 446 and 372 DEGs were identified in females treated with PFOA and PFOS, respectively, while 327 and 329 DEGs were identified in similarly treated males (Figures 4B, S5C, and S5D). This progressive increase in the number of DEGs with dose and exposure duration underscores the cumulative impact of PFAS on hepatic gene regulation.

To explore the functional implications of these transcriptional changes, KEGG pathway enrichment analysis was performed. For the 28-day cohort, DEGs were filtered using the same threshold (FC > 1.5 and a *p*_adj_ < 0.05). For the 56-day cohort, DEGs were identified based on BMD modeling criteria. Specifically, genes were selected if they met one or both of the following thresholds—(1) a BMD upper-to-lower CI ratio (BMDU/BMDL) ≥40, and/or (2) a statistically significant best-fit model *p* value (≤0.05) (Figure S5E)—to target DEGs that exhibit a dose-responsive effect. KEGG pathways were then discriminated by *q* value < 0.05. The top 10 enriched KEGG pathways revealed a high degree of overlap across sex, time point, and PFAS treatment groups, indicating a set of commonly affected biological processes. Notably, PPAR signaling pathway (mmu03320) was identified as a top 10 enriched pathway across all conditions. Major genes related to this pathway include *P*para and *P*parg, which are upregulated in several conditions following PFAS exposure. Several pathways related to lipid metabolism were also identified, including fatty acid degradation (mmu00071), steroid hormone biosynthesis (mmu00140), fatty acid metabolism (mmu01212), arachidonic acid metabolism (mmu00590), linoleic acid metabolism (mmu00591), and biosynthesis of unsaturated fatty acids (mmu01040), which all indicate a broad disruption of lipid metabolism (Figure 4B).

To compare pathway-level enrichment between sexes and PFAS type, KEGG enrichment analyses were aggregated across exposure duration (28- and 56-days) and sexes (male and females) or PFAS exposures (PFOA and PFOS). For each pathway, the minimum FDR-adjusted *q* value across all conditions was used to represent the strongest evidence of enrichment. Female and male (Figure 4C) as well as PFOA and PFOS (Figure 4D) enrichment profiles were then compared in scatter plots of *q* values. Overall, pathway enrichment patterns were highly similar between sexes and PFAS type, as reflected by a strong positive correlation between *q* values (Figures 4C and 4D).

### PFOA- and PFOS-induced sex-specific immunotoxicity

**Figure 5:**
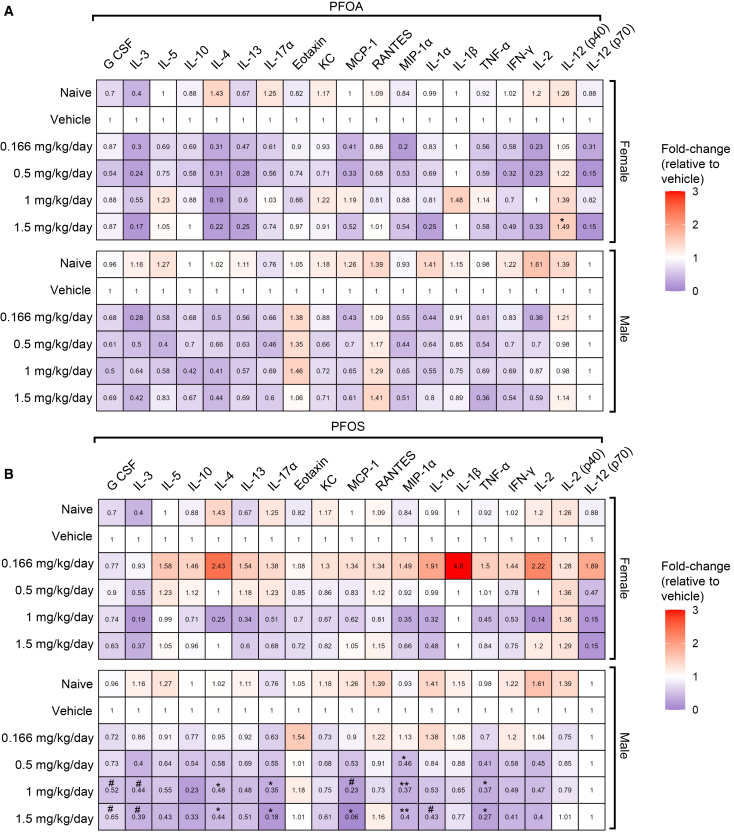
PFOA and PFOS cause dysregulation of plasma cytokines Median-fold change (vs. same-sex vehicle) of 19 cytokines at euthanasia following exposure to PFOA (A) or PFOS (B) *n* = 8 mice/group. Statistical significance was assessed using the Kruskal-Wallis test with Dunn’s multiple comparisons (control: vehicle) for each parameter. Data are presented as median fold change. #*p* ≤ 0.099; ∗*p* ≤ 0.05; ∗∗*p* ≤ 0.01; ∗∗∗*p* ≤ 0.001. CCL5 (RANTES), C-C motif chemokine ligand 5; G-CSF, granulocyte colony-stimulating factor; IFN-γ, interferon-gamma; IgM, immunoglobulin M; IL, interleukin; KC, keratinocyte chemoattractant; MCP-1, monocyte chemoattractant protein-1; MIP, macrophage inflammatory protein; PFOA, perfluorooctanoic acid; PFOS, perfluorooctanesulfonic acid; SRBC, sheep red blood cells; TNF-α, tumor necrosis factor-alpha.

Next, to evaluate the immunotoxic effects of PFOS and PFOA exposure, experimental mice (excluding naive controls) were injected with sheep red blood cells (SRBCs) 5 days prior to euthanasia (Figures 1A, S6A, and S6B). A panel of 19 cytokines was measured in mouse plasma to broadly assess immune function (Figures S6A and S6B). PFOA exposure resulted in a modest but non-significant decrease in several cytokines across multiple doses. The only significant change observed was in interleukin (IL)-12 (p40), which increased significantly in female mice at 1.5 mg/kg/day. PFOA-exposed male mice also showed modest decreases in several cytokines, but no significant changes were observed (Figures 5A and S6C). PFOS exposure significantly reduced plasma levels of IL-4, IL-17α, monocyte chemoattractant protein-1 (MCP-1), macrophage inflammatory protein (MIP)-1α, and tumor necrosis factor-alpha (TNF-α) in male mice, with effects observed at doses as low as 0.5 mg/kg/day. Additionally, non-significant decreases were also observed in granulocyte colony-stimulating factor (G-CSF), IL-3, and IL-1α starting at 1 mg/kg/day. In females, PFOS had a minimal effect, with no cytokine showing significant changes at any dose (Figures 5B and S7A). These findings suggest a sex-specific immunotoxic response to PFOS, with males exhibiting greater cytokine suppression compared to females. Other cytokines measured, including IL-5, 1L-10, IL-13, eotaxin, keratinocyte chemoattractant (KC), Regulated Upon Activation, Normal T-cell Expressed and Secreted (RANTES), IL-1β, interferon-gamma (IFN-γ), IL-2, and IL-12 (p70), presented no significant change from the vehicle group (Figures 5A, 5B, S6, and S7).

**Figure 6:**
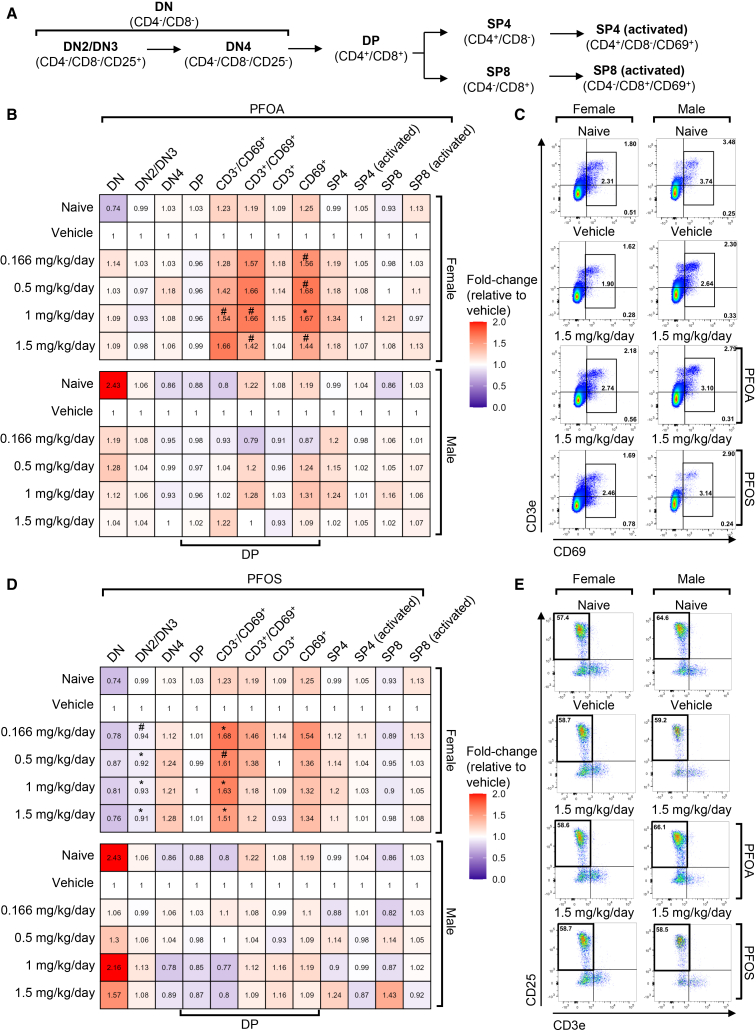
PFOA and PFOS cause changes in thymocyte development in female mice (A) Schematic of thymocyte development in the thymus. (B) Median-fold change (vs. same-sex vehicle) of thymus cell populations at euthanasia following exposure to PFOA. (C) Representative dot plots gated on DP cells for CD3e/CD69 staining naive, vehicle, 1.5 mg/kg/day PFOA, and 1.5 mg/kg/day PFOS-treated males and females. Black box shows CD69^+^ cells. (D) Median-fold change (vs. same-sex vehicle) of thymus cell populations at euthanasia following exposure to PFOS. (E) Representative dot plots gated on DN cells for CD25/CD3e staining in naive, vehicle, 1.5 mg/kg/day PFOA, and 1.5 mg/kg/day PFOS-treated males and females. Black box shows DN2/DN3 cells. *n* = 7–8 mice/group. Statistical significance was assessed using the Kruskal-Wallis test with Dunn’s multiple comparisons (control: vehicle) for each parameter. Data are presented as median fold change. #*p* ≤ 0.099; ∗*p* ≤ 0.05. DN, double-negative (CD4^-^/CD8^-^) thymocytes; DN2/DN3, double-negative stage 2/3 (CD4^-^/CD8^-^/CD25^+^) thymocytes; DN4, double-negative stage 4 (CD4^-^/CD8^-^/CD25^-^) thymocytes; DP, double-positive (CD4^+^/CD8^+^) thymocytes; PFOS, perfluorooctanesulfonic acid; PFOA, perfluorooctanoic acid; SP4, single positive (CD4^+^/CD8^-^) T cells; SP8, single positive (CD4^-^/CD8^+^) T cells.

To further investigate the immunotoxicity caused by PFOA and PFOS exposure, key immune organs were collected for flow cytometry analysis, including the thymus (a primary immune organ) and the spleen (a secondary immune organ). The thymus, a central site for thymocyte development, was assessed for shifts in developmental stages of thymocytes, including double-negative (DN; CD4^-^/CD8^-^), double-positive (DP; CD4^+^/CD8^+^), single-positive (SP4) CD4^+^, and single-positive CD8^+^ (SP8) subsets (Figures 6A and S8A). Two specific stages of DN cell development are highlighted here, which are DN2/DN3 and DN4. Importantly, DN4 markers, CD4^-^/CD8^-^/CD25^-^, could also characterize the DN1 population in the thymus. However, since DN1 cells represent 0.01% of the total progenitor population present in the thymus, this population has been labeled as the more abundant DN4 T cell population.^41^ Due to interest in the DP population, further subsets of these cells were assessed, including pre-selected DP (CD3^-^/CD69^+^), positively selected DP (CD3^+^/CD69^+^), and resting DP (CD3^+^/CD69^-^). These stages reflect the progression from early progenitor cells to mature T cells capable of peripheral function (Figure S8A).

In females exposed to PFOA, a slight increase in pre-selected DP cells (*p* = 0.06) and positively selected DP cells (*p* = 0.06) was detected starting at 1 mg/kg/day (Figures 6B, 6C, and S9A). Similarly, in PFOS-exposed females, the percentage of pre-selected DP cells was significantly elevated starting at 0.5 mg/kg/day, and DN2/DN3 cells were significantly reduced at the same dose (Figures 6D, 6E, and S9B). These findings suggest a female-specific disruption in thymocyte development, characterized by impaired progression through early DN stages and increased accumulation of DP cells. No changes in total DN, DN4, DP, resting DP, CD3^+^, SP4, SP4 (activated), SP8, or SP8 (activated) were observed. Furthermore, other committed cell types, including macrophages, dendritic cells, and B cells, were also unchanged (Figures 6A–6E, S9A, and S9B).

The spleens of PFAS-exposed mice were assessed for changes in total T cells, SP4, SP8, B cells, and dendritic cells (Figure S8B). No significant changes in splenic immune cell populations were observed in any treatment group (Figures S10A–S10D).

To investigate a key site for immune communication and mobilization, the blood of PFOA and PFOS-exposed mice was collected at euthanasia and analyzed for major immune cell populations, including total T cells, SP4, SP8, B cells, and monocytes. PFOA exposure over 56 days caused an increase in total T cell populations at 1.5 mg/kg/day in male mice only; however, this trend did not reach significance at 95% CI (*p* = 0.06) (Figures S11A–S11C and S12A–S12C).

## Discussion

This study investigated the temporal and sex-specific effects of PFOA and PFOS on *in vivo* mouse hepatic and immunologic functions. By integrating organ weight, serum biomarkers, transcriptomics, flow cytometry, and cytokine profiling, we identified overlapping and distinct patterns of toxicity in male and female mice across the hepatic and immune system, consistent with previous research, while highlighting additional novel insights.

A key finding from this study is the distinct toxicokinetic features of PFOA and PFOS. Specifically, we found that despite being issued at the same concentration, PFOA accumulates to a much lower extent than PFOS in the liver and plasma. We suspect this is likely due to its faster elimination and lower affinity for plasma proteins compared to PFOS.^38^^,^^39^^,^^40^ These differences are further highlighted by our time-course data. At the 28-day time point, the average PFOS concentration in plasma was 135.53 mg/L, which increased to 199.69 mg/L by day 56. In contrast, PFOA concentrations rose only slightly, from 50.49 mg/L at 28 days to 55.27 mg/L at 56 days. This suggests that PFOS had not yet reached steady state by day 56, whereas PFOA had. These divergent accumulation patterns underscore how compound-specific toxicokinetics shape internal exposure levels, with implications for downstream biological effects.

Consistent with prior studies, exposure to PFOA and PFOS led to dose-dependent increases in liver-to-body weight ratios in both sexes following 28 and 56 days of exposure. This hepatomegaly was accompanied by elevated levels of serum biomarkers associated with hepatocellular and hepatobiliary dysfunction, such as ALP and ALT, which increased significantly in both sexes. These findings align with previous reports showing PFAS-induced liver enlargement, and elevated serum ALT and ALP levels.^24^^,^^25^^,^^26^^,^^27^^,^^28^^,^^29^^,^^30^^,^^31^ In contrast, serum TGL levels decreased in a dose-dependent manner, suggesting disruption of lipid metabolism. This aligns with previous studies that have shown PFOA results in decreased plasma TGL levels^42^^,^^43^ while increasing hepatic TGL accumulation, leading to liver toxicity.^44^

The observed liver enlargement and altered serum biomarkers are consistent with PPAR-mediated mechanisms of PFAS-induced hepatotoxicity. PFASs, particularly PFOA and PFOS, are known ligands for PPARs, especially PPARα and PPARβ/δ. This regulates genes involved in lipid metabolism, fatty acid oxidation, and hepatocyte proliferation.^45^ Chronic activation of PPARα in rodents has been shown to induce hepatomegaly through both peroxisome proliferation and hypertrophy.^42^ Our transcriptomic analysis revealed significant enrichment of the KEGG PPAR signaling pathway (mmu03320) across both sexes and time points following exposure to PFOS and PFOA, suggesting potential PPAR involvement. In addition, multiple lipid metabolism-related pathways were enriched in exposed animals, further supporting a role for disrupted lipid metabolism. This is supported by previously published work which found that liver spheroids treated with several PFASs, including PFOS and PFOA, had disrupted CHOL synthesis and uptake.^46^

Conversely, research has also shown that exposure to high-dose PFOA increased liver weight in both wild-type and PPARα^−/−^ mice, while reductions in plasma TGL occurred only in PPARα-competent animals. This pattern indicates that hepatomegaly at high doses is largely PPARα-independent, whereas the TGL-lowering effect is PPARα-dependent.^42^ Other mechanisms such as constitutive androstane receptor (CAR) and PXR are likely involved.^42^^,^^47^ Together, these results point to multiple mechanisms of PFAS hepatotoxicity.^42^ Moreover, it has been shown that using mice expressing human PPARα mice alleviates the decreased serum TGL observed in mice expressing mouse PPARα, implying limited inter-species translatability.^48^ Together, these findings reinforce the view that PFAS-induced liver toxicity may in part arise from nuclear receptor activation, but protein-level and functional validation are still required to confirm this.

Beyond hepatic effects, a significant reduction in pancreas-to-body weight ratios was also observed, particularly in females at 28 days and males at 56 days following exposure to 1.5 mg/kg/day PFOS or PFOA. Multiple previous studies have observed alterations in pancreatic function and structure. Qin et al. previously observed reduced pancreas size, impaired islet cell function, and decreased insulin levels following PFOS exposure, consistent with increased diabetes risk^8^ or insulitis.^49^

Elevated serum levels of amylase and lipase, which are typically observed in conjunction with decreased insulin secretion, have also been observed following PFOA treatment in mice.^7^^,^^50^ Typically, hypertriglyceridemia is observed in diabetes patients; however, in combination, these findings suggest a more complex mechanism that requires further investigation. Ultimately, changes in liver and pancreas weights, along with clinical chemistry endpoints, appeared largely independent of PFAS type, sex, or exposure duration.

Our transcriptomic BMD analysis also revealed a sex-specific pattern of potency, which showed that the top 10 most sensitive genes were associated with PFOA exposure in females and with PFOS exposure in males. This suggests that PFOA was more potent in female mice, whereas PFOS showed greater potency in males. These findings are consistent with previous developmental studies reporting greater PFOS sensitivity in male pups compared to females.^51^

Interestingly, while sex-specific differences in hepatic injury markers were minimal, there was a clear divergence in immune-related outcomes. Measurement of 19 cytokines identified broad suppression of both pro-inflammatory and regulatory cytokines in males following PFOS exposure. This included reductions in IL-4, IL-17α, TNF-α, and MCP-1, beginning at 0.5 mg/kg/day. Paradoxically, males also showed a non-significant increase in T cell populations following PFOA exposure, potentially reflecting compensatory proliferation or altered T cell trafficking in response to exposure. Notably, the modest increase in peripheral T cells observed in PFOA-treated males could not be linked to thymic changes, raising the possibility of divergent mechanisms across sexes.

In contrast to males, females exhibited fewer changes in cytokine profiles. The most prominent effect was a significant increase in IL-12(p40) following PFOA exposure, a cytokine primarily implicated in negative feedback regulation through its interaction with the IL-12 receptor.^52^ The most notable immunotoxic effects in females were observed in the thymus. Flow cytometry revealed alterations in thymocyte development following PFAS exposure. Specifically, PFOS significantly increased the number of pre-selected DP thymocytes (CD4^+^/CD8^+^/CD3^-^/CD69^-^), a population of immature thymocytes prior to T cell receptor (TCR) engagement. PFOA exposure led to a modest increase in both pre-selected DP cells and positively selected DP cells (CD4^+^/CD8^+^/CD3^+^/CD69^+^), which are transitioning toward single positive (SP) T cells following successful TCR signaling. These changes in early (pre-selection) and intermediate (positive selection) thymocyte subsets suggest that PFOS and PFOA may differentially disrupt thymic T cell maturation processes, potentially altering peripheral T cell generation. However, it is critical to note that despite being administered at the same oral dose, PFOA accumulates to a much lower extent than PFOS in the plasma and liver, indicating a difference in pharmacokinetic parameters such as absorption, distribution, metabolism, or excretion. A difference in bioavailability may be responsible for the PFAS-specific decreases in circulating cytokines that we observed in this study.

Our findings emphasize the importance of examining sex-specific effects, as earlier studies conducted exclusively in male mice reported decreased DP thymocytes following PFOA treatment.^53^^,^^54^ As addressed above, these findings support the idea of divergent mechanisms across sexes. Specifically, males may exhibit compensatory T cell production following PFAS exposure, while females may directly promote T cell production independently of peripheral cues, leading to immunologic dysfunction. Since PFASs can act as endocrine disruptors and thymocytes are sensitive to sex hormone signaling, female thymocytes may be particularly susceptible to PFAS-induced dysregulation.^55^^,^^56^ However, future studies are required to confirm the exact mechanism by which this occurs. Moreover, while we observed immune perturbations in our SRBC-immunized model, it is important to note that other studies using naive mice and more human-relevant PFAS doses have reported minimal effects on immune cell populations and serum antibody production.^57^ Thus, our findings highlight that PFOA exposure in the context of an active immune challenge may have biological impacts that are not apparent under unchallenged conditions.

Several molecular mechanisms may underlie the observed immunotoxic effects. PPARs, NF-κB, and Ca^2+^ signaling have all been implicated in PFAS-induced immunotoxicity.^23^ PPAR signaling has been implicated in both hepatic and immune outcomes and was enriched in several treatment groups as previously described. PPAR may be a critical mediator between these two PFAS-targeting systems. Recent studies have found that macrophages, a key component of the hepatic immune system, are important targets of PFAS-induced lipid accumulation, partially via PPARγ activation.^58^ You et al. further showed that PFAS suppress macrophage alternative activation, disrupting lipid metabolism via PPARγ and PPARα.^59^ Collectively, these findings suggest that immune cells within the liver are directly affected by PFAS-induced lipid dysregulation and may mediate downstream immuno-impairment, potentially toward anti-inflammation.^60^

Apart from PPAR signaling, additional immune-relevant pathways emerged from our transcriptomic analysis. Notably, significant enrichment of the advanced glycation end product (AGE)-receptor for (RAGE) signaling pathway linked to diabetic complications (mmu04933) was identified across multiple groups. Although traditionally associated with diabetes, this pathway’s core mechanism, the binding of AGEs to their receptor RAGE, initiates a signaling cascades that leads to the activation of NF-κB.^61^ This cascade promotes oxidative stress, cytokine production, and immune cell activation, all of which are consistent with known PFAS-induced immune perturbations. Thus, the enrichment of the AGE-RAGE-NF-κB axis suggests that PFAS exposure may contribute to immune dysfunction via inflammatory signaling. However, direct measurements of this proposed pathway are absent from the current study and require further investigation.

Overall, this study presents three key and novel findings. First, we report that both PFOA and PFOS induce not only hepatomegaly, a well-established outcome, but also pancreatic atrophy, a less recognized target of PFAS toxicity. Second, we show that PFAS-induced hepatic toxicity occurs independently of sex. Finally, we reveal striking sex-specific immune perturbations. Here, male mice exhibited broad suppression of cytokines and altered peripheral T cell populations, whereas females showed targeted disruptions in thymocyte development. These findings highlight sex as a critical, yet underexplored, variable in PFAS-induced immunotoxicity and emphasize the need for mechanistic studies to dissect the sex-specific pathways driving immune dysregulation.

### Limitations of the study

We acknowledge that this study has several limitations. First, the asymmetrical dosing design limits direct comparison with the 56-day study. This was selected to align with common 28-day designs while considering ethical and financial constraints. Second, estrous cycle monitoring was not performed, which may confound the interpretation of sex-specific immune responses. Third, the group size (*n* = 8/group/sex) provided adequate power for key endpoints but may limit sensitivity for subtle sex-dependent effects. Fourth, differences in housing between sexes may have introduced stress-related variability, potentially influencing immune and metabolic outcomes and the interpretation of sex-based comparisons. Fifth, a positive control for the TDAR assay was not included, which limits the ability to confirm assay sensitivity. Finally, confirmatory histopathology for liver and pancreatic alterations, pancreatic enzyme analysis, and qPCR for altered gene expression was not conducted and represents an important next step for validating these mechanistic findings.

## Resource availability

### Lead contact

Correspondence should be addressed to the lead contact, Azam Tayabali (azam.tayabali@hc-sc.gc.ca).

### Materials availability

This study did not generate new unique reagents. The materials underlying this article will be shared upon reasonable request to the lead contact.

### Data and code availability

• Transcriptomics data have been deposited at Gene Expression Omnibus (GEO) and are publicly available as of the date of publication. Accession numbers are listed in the key resources table.
• All code has been deposited at GitHub and is publicly available as of the date of publication. Any additional information required to reanalyze the data reported in this paper is available from the lead contact upon request.
• Raw data from Figures 1, 2, 3, 5, and 6 were deposited on Mendeley at https://doi.org/10.17632/vfbcx4zndf.1.

## Acknowledgments

This work was funded by the Water and Air Quality Bureau (WAQB) within the Healthy Environments and Consumer Safety Branch of Health Canada. We thank members of the Scientific Services Division (SSD) of Health Products and Food Branch (HPFB) of Health Canada, Brittany Parks, Nadine Busselot, Stephanie Keith-Tallon, Karine Chamberland, Gen Sheng Wang, Michelle Lalande, and Dr. Alyssa Calder, for their invaluable support with animal care, necropsy, and study coordination. We also thank Anna Yip for her assistance with tissue processing and sample preparation for flow cytometry. Their contributions were crucial to the project’s success. The graphical abstract was created in BioRender. Loan, A. (2026) https://BioRender.com/q5jmj81.

## Author contributions

A.B. performed chemical treatments, tissue isolation and preparation, flow cytometry sample preparation, and ELISA experiments; A.L. performed data analysis and figure preparation; A.W. contributed to the chemical treatments, solution preparation, and sample preparation related to flow cytometry and ELISA experiments; H.M. contributed to tissue isolation and flow cytometry sample preparation; D.P. performed flow cytometry gating and data analysis; M.J.M., E.C., and L.M.B. performed bioinformatic analysis for transcriptomic analysis; E.C. performed sequencing library preparation and BMD analysis; Z.G. performed LC-MS/MS analysis of mouse plasma and liver samples; G.P., A.N., R.A-R., K.M.E., and A.T. contributed to the conceptualization, funding, and project management; A.B., A.L., G.P., M.N., M.J.M., A.N., R.A-R., K.M.E., D.P., and A.T. contributed to experimental design and writing the paper.

## Declaration of interests

The authors declare no competing interests.

## Declaration of generative AI and AI-assisted technologies in the writing process

During the preparation of this work, the authors used ChatGPT to assist with editing, condensing, and clarifying the text. All content was subsequently reviewed and further edited by the authors.

## STAR★Methods

### Key resources table

**Table:** 

REAGENT or RESOURCE	SOURCE	IDENTIFIER
**Antibodies**		

BD Horizon™ Fixable Viability Stain 510	BD	Cat#564406; RRID:AB_2869572
BD Pharmingen™ Purified Rat Anti-Mouse CD16/CD32 (Mouse BD Fc Block™)	BD	Cat#553142; RRID:AB_394657
BD Horizon™ Brilliant Stain Buffer Plus	BD	Cat#566385; RRID:AB_2869761
BUV395 Rat Anti-Mouse CD4	BD	Cat#563790; RRID:AB_2738426
BUV737 Rat Anti-Mouse CD335 (NKp46)	BD	Cat#612805; RRID:AB_2870131
BV421 Hamster Anti-Mouse CD11c	BD	Cat#562782; RRID:AB_2737789
BV786 Rat Anti-Mouse CD45R/B220	BD	Cat#563894; RRID:AB_2738472
FITC Hamster Anti-Mouse CD3e	BD	Cat#553061; RRID:AB_394594
PE Hamster Anti-Mouse CD69	BD	Cat#561932; RRID:AB_10897992
PE-CF594 Rat Anti-Mouse F4/80	BD	Cat#565613; RRID:AB_2734770
PE-Cy™ 7 Rat Anti-Mouse CD25	BD	Cat#552880; RRID:AB_394509
PerCP-Cy™ 5.5 Rat Anti-Mouse CD45	BD	Cat#550994; RRID:AB_394003
R718 Rat Anti-Mouse CD8a	BD	Cat#566985; RRID:AB_2869989

**Biological samples**		

Mouse: Blood and Plasma (K2 EDTA-Treated)	This paper	–
Mouse: Gastrointestinal (GI) tract (esophagus to rectum)	This paper	–
Mouse: Kidneys	This paper	–
Mouse: Kidneys	This paper	–
Mouse: Liver	This paper	–
Mouse: Pancreas	This paper	–
Mouse: Serum	This paper	–

**Chemicals, peptides, and recombinant proteins**		

Tween 20	Millipore Sigma	Cat#P1379
Water, Optima™ LC/MS Grade	Thermo Fisher Scientific	Cat#W64
Perfluorooctanoic acid (PFOA)	Millipore Sigma	Cat#171468
Perfluorooctanesulfonic acid (PFOS)	Millipore Sigma	Cat#77283
Methanol	Thermo Fisher Scientific	Cat#047192-K2
Mass-Labelled PFAS Extraction Standard Solution (Internal Standard Mix; IS)	Wellington Laboratories	Cat#MPFAC-24ES
Formic Acid	Sigma-Aldrich	Cat#27001-1L-R
Ammonium acetate	Fisher Scientific	Cat#A639-500
Sheep Red Blood Cells (SRBC) 10% Washed Pooled Cells	Rockland	Cat#R405-0050

**Critical commercial assays**		

Alkaline Phosphatase Reagent Cartridge	Siemens	Cat#10642445
Alanine Aminotransferase Reagent Cartridge	Siemens	Cat#10475530
Calcium Reagent Cartridge	Siemens	Cat#10444949
Cholesterol Reagent Cartridge	Siemens	Cat#10444891
Lactate Dehydrogenase Reagent Cartridge	Siemens	Cat#10284483
Free Thyroxine (FT4L) Reagent Cartridge	Siemens	Cat#10464524
Total Bilirubin Reagent Cartridge	Siemens	Cat#10444957
Triglycerides Reagent Cartridge	Siemens	Cat#10444906
Chemistry I Multiple Analytes Calibrator	Siemens	Cat#10716280
Chemistry II Multiple Analytes Calibrator	Siemens	Cat#10444997
Enzyme I Calibrator	Siemens	Cat#10284680
Enzyme II Calibrator	Siemens	Cat#10476170
Alkaline Phosphatase Calibrator	Siemens	Cat#10714028
Cholesterol Calibrator	Siemens	Cat#10444998
Total Bilirubin/Direct Bilirubin Calibrator	Siemens	Cat#10445013
RNeasy Mini Kit	QIAGEN	Cat#74104
RNase-Free DNase Set	QIAGEN	Cat#79254
TempO-Seq Mouse S1500+ Gene Panel (v1.2)	BioSpyder Technologies	Cat# KT-06-096
KAPA SYBR FAST Universal qPCR Kit	Roche	Cat#07960140001
Mouse Anti-SRBCs IgM ELISA Kit	Life Diagnostics	Cat#SRBCM-1
Bio-Plex Mouse Cytokine Group 1 Panel 23-Plex	Bio-Rad	Cat#M60-009RDPD
Spleen Dissociation Kit, Mouse	Miltenyi	Cat#130-095-926
10X RBC Lysis Buffer	Invitrogen	Cat#00-4300-54
BD Pharmingen™ Transcription Factor Buffer Set	BD	Cat#562574
UltraComp eBeads™ Compensation Beads	Invitrogen	Cat#01-2222-42
ArC™ Amine Reactive Compensation Bead Kit	Invitrogen	Cat#A10346

**Deposited data**		

Transcriptomic data	This paper	https://www.ncbi.nlm.nih.gov/geo/; accession number: GSE303480
R script for data analysis	This paper	https://github.com/HC-EHSRB-CompTox
Raw data	This paper	Mendeley data: https://doi.org/10.17632/vfbcx4zndf.1

**Experimental models: Organisms/strains**		

Mouse: C57BL/6-Elite (SOPF) mouse	Charles River	Cat#475C57BL/6-E

**Software and algorithms**		

bcl2fastq (v2.20)	Illumina	https://support.illumina.com/sequencing/sequencing_software/bcl2fastq-conversion-software.html; RRID:SCR_015058
Omics Data Analysis Frameworks for Regulatory application (R-ODAF) Health Canada pipeline	Verheijen et al.^62^	https://github.com/R-ODAF/R-ODAF_Health_Canada
Snakemake (v8.0)	Mölder et al.^63^	https://bitbucket.org/johanneskoester/snakemake/wiki/; RRID:SCR_003475
Fastp (v0.23.2)	Chen^64^	https://github.com/OpenGene/fastp; RRID:SCR_016962
STAR (v2.7.8a)	Dobin et al.^65^	http://code.google.com/p/rna-star/; RRID:SCR_004463
QuasR R Package (v1.30.0)	Gaidatzis et al.^66^	https://www.bioconductor.org/packages/release/bioc/html/QuasR.html
DESeq2 R Package (v1.38.0)	Love et al.^67^	https://bioconductor.org/packages/release/bioc/html/DESeq2.html
EPA BMDS Software	US EPA^68^^,^^69^	https://bmdsonline.epa.gov/
BMDExpress3	Division of Translational Toxicology (National Institute of Environmental Health Sciences)	https://github.com/auerbachs/BMDExpress-3
FlowJo (v10.9.0)	BD	https://www.flowjo.com/; RRID:SCR_008520
R (v4.3.2)	R Core Team	https://www.r-project.org/; RRID:SCR_001905

**Other**		

PowerBead Tubes, Metal 2.38 mm	QIAGEN	Cat#13117-50
WAX μSPE Cartridges (30 mg)	ITSP Solutions	Cat#30P-WOWAX-T
TSQ Altis Plus Triple Quadrupole MS	Thermo Fisher Scientific	https://www.thermofisher.com/order/catalog/product/TSQ03-10002
Vanquish UPLC System	Thermo Fisher Scientific	https://www.thermofisher.com/ca/en/home/industrial/chromatography/liquid-chromatography-lc/hplc-uhplc-systems/vanquish-flex-uhplc-system.html
ACQUITY UPLC BEH C18 Column (1.7 μm, 2.1 × 50 mm)	Waters	Cat#186002350
VanGuard BEH C18 Pre-column (1.7 μm, 2.1 × 5 mm)	Waters	Cat#186003975
Isolator Column (2.1 × 50 mm)	Waters	Cat#186004476
Dimension EXL 200 Integrated Chemistry System	Siemens	https://www.siemens-healthineers.com/en-us/laboratory-diagnostics/clinical-chemistry-and-immunoassay-systems/dimension-exl-200-integrated-chem-sys
PowerBead Tubes, Ceramic 2.8 mm	QIAGEN	Cat#13114-50
NextSeq 500 Sequencer	Illumina	https://support.illumina.com/sequencing/sequencing_instruments/nextseq-500.html
Bio-Plex 200 System	Bio-Rad	https://www.bio-rad.com/en-ca/product/bio-plex-200-systems?ID=715b85f1-6a4e-41b3-b5d9-80202d779e13
gentleMACS Octo Dissociator with Heaters	Miltenyi	https://www.miltenyibiotec.com/CA-en/products/gentlemacs-octo-dissociator-with-heaters.html#130-134-029

### Experiment model and study participant details

#### Animals and housing

All animal protocols were approved by the Health Canada Animal Care Committee (HC-ACC protocol# 2023-010). All experiments were done in accordance with the Canadian Council on Animal Care standards.

6 to 9-week-old male and female C57BL/6-Elite (SOPF) mice (Charles River, cat#475C57BL/6-E) were housed in Tecniplast IVC cage system with a 12:12 light cycle. Mice were fed irradiated Teklad Global 14% protein rodent diet (Inotiv, cat#2914) *ad libitum* and provided autoclaved reverse osmosis water in Tecniplast bottles (Table S1). After a two-week acclimatization period, the experimental methodology began. This age range is commonly used to represent young adult mice in immunological studies, as many immune parameters (e.g., B cells, T cells, and hematopoietic progenitor cells) are fully developed and exhibit adult-like functional profiles at this stage.^70^^,^^71^^,^^72^ While metabolic development continues beyond this age, variability is significantly reduced relative to younger mice.^72^

Throughout the study, mice were housed individually (male) or in pairs (female). While group housing is generally preferred due to the social nature of mice, male mice are known to be territorial, which increases the risk of aggression, injury and associated variability in immune response and welfare outcomes.^73^^,^^74^ To mitigate these risks, males were housed individually. Females, on the other hand, are more tolerant and were group-housed to avoid isolation-induced stress, optimize welfare and housing efficiency.^73^

#### Group size and randomization

A group size of eight mice per group (*n* = 8) was determined based on power calculations from preliminary range-finding experiments (data not shown), while also considering ethical principles (3Rs). A one-way ANOVA sample size calculator was used for the sample size estimation (https://homepage.univie.ac.at/robin.ristl/samplesize.php?test=anova).

Mice were randomly assigned to experimental groups using the standard “ = RAND()” function in Microsoft Excel (v2508), in accordance with the Animal Research: Reporting of *In Vivo* Experiments (ARRIVE) guidelines. Group assignments were then adjusted to ensure comparable mean body weights and balanced age distributions across groups. Specific stages of the study were conducted in blinded conditions, as recommended by ARRIVE guidelines. Experimental procedures were not carried out in a blind manner to reduce potential staff exposure. However, tissue collection, data acquisition, and data analysis were performed blindly.^75^

### Method details

#### Compound preparation and administration

At 8 to 11 weeks of age, mice were exposed to 1.5 mg/kg/day of perfluorooctanoic acid (PFOA) or perfluorooctanesulfonic acid (PFOS) for 28 days or 0.166, 0.5, 1.0, or 1.5 mg/kg/day of PFOA or PFOS for 56 days by daily oral gavage (in a constant gavage volume of 7.5 mL/kg). Briefly, Tween 20 (Millipore Sigma, cat#P1379) was diluted in optima water (Thermo Fisher Scientific, cat#W64) to obtain a 0.5% (v/v) solution. PFOA (Millipore Sigma, cat#171468) or PFOS (Millipore Sigma, cat#77283) was added to the 0.5% Tween 20 solution to achieve a 1.5 mg/kg/day gavage solution. The 1.0, 0.5, and 0.166 mg/kg/day gavage solutions were obtained through serial dilution. The vehicle groups were gavaged with 0.5% Tween 20 in optima water, while the naive groups were not gavaged.

PFOA and PFOS were studied in parallel because they represent two major PFAS subclasses (carboxylates and sulfonates) and remain the most frequently detected PFAS in human biomonitoring, allowing assessment of structural differences that may influence immune and hepatic toxicity. A single high dose (1.5 mg/kg/day) was selected for the 28-day study to provide a benchmark consistent with published 28-day studies, while a wider range of doses (0.166–1.5 mg/kg/day) was used for the 56-day study to enable dose–response assessment. While the selected doses are higher than typical human environmental exposures, they were informed by previously reported lowest-observed-adverse-effect level (LOAEL) from animal studies^9^: ∼3.75 mg/kg/day for PFOA immune effects, 0.00166–0.8 mg/kg/day for PFOS immune effects, ∼1 mg/kg/day for PFOA liver effects, and ∼3 mg/kg/day for PFOS liver effects. Since our exposures (28 and 56 days) were longer than many of the studies informing these LOAELs, we selected lower nominal doses to account for higher accumulated internal dose over time while still ensuring biologically relevant responses.

#### Sample collection

Body weights were measured daily, and water consumption was measured weekly. Water consumption was calculated by measuring the difference in weight between a full water bottle and the same bottle after one week of use.

Mice treated for 28 or 56 days were euthanized by exsanguination (cardiac puncture) under isoflurane anesthesia 24 h following the last oral gavage. Blood was collected by cardiac puncture in serum separator tubes (BD, cat#365967) and potassium ethylenediaminetetraacetic acid (K2 EDTA) blood collection tubes (BD, cat#365974). Additionally, the liver, pancreas, kidneys, and gastrointestinal (GI) tract (esophagus to rectum) were collected and weighed.

#### Quantification of PFAS in the plasma and liver

All samples were stored at −80 °C until analysis. For liver samples, frozen tissue was cut using a disposable blade and accurately weighed (40 ± 10 mg) into 2.0 mL PowerBead tubes (Qiagen, cat#13117-50). Each sample was homogenized in 1.0 mL of 70% LC-MS grade methanol (Thermo Fisher Scientific, cat#047192-K2) by using a PowerLyzer 24 Homogenizer (Qiagen) at 3500 rpm for 45 s. The homogenates were then sonicated for 20 min and centrifuged at 10,000 rpm for 10 min. The range in concentration between control and high-dose exposure extends for around three orders of magnitude; therefore, control samples and exposed samples were processed differently. For control (naive and vehicle) liver samples, 250 μL (∼10 mg tissue) of the resulting supernatant was spiked with 10 μL of Internal Standard (IS) mixture (Wellington Laboratories, cat#MPFAC-24ES) (25 ng/mL) and 750 μL of 60% methanol containing 2% formic acid (Sigma-Aldrich, cat#27001-1L-R). The mixture was then vortexed and subjected to micro-solid phase-extraction (μSPE) clean-up. Supernatants from exposed liver samples were processed using a dilute-and-shoot (DS) approach, with an appropriate dilution factor using methanol as the solvent. The IS mixture was added to the final dilution at a concentration of 2.5 ppb prior to liquid chromatography-tandem mass spectrometry (LC-MS/MS) analysis.

Plasma samples were thawed at room temperature. For control groups, 10 μL of plasma was mixed with 10 μL of IS mixture (25 ng/mL) and 980 μL of 60% methanol containing 2% formic acid, followed by 20 min sonication and μSPE extraction. For exposed groups, 10 μL of plasma was transferred to a 1.5 mL polypropylene centrifuge tube and mixed with 990 μL of 80% methanol. Samples were sonicated for 20 min and centrifuged at 10,000 rpm for 10 min. The resulting supernatants were appropriately diluted with methanol containing the IS mixture at a final concentration of 2.5 ppb prior to LC-MS/MS analysis.

The μSPE sample cleanup was conducted using 30 mg WAX μSPE cartridges (ITSP Solutions, cat#30P-WOWAX-T) with full automation via the PAL-RTC system (CTC Analytics AG, Switzerland). A total volume of 950 μL of acidified extract was loaded onto the pre-cleaned cartridge at a rate of 5 μL/s, followed by two sequential washes. PFAS were eluted using 900 μL of 20 mM high-performance liquid chromatography (HPLC) grade ammonium acetate (Fisher Scientific, cat#A639-500) in methanol. Eluates were evaporated to dryness using a TurboVap system at 45 °C with a nitrogen flow rate of 0.7 mL/min. The dried extracts were reconstituted in 95 μL methanol and transferred to a 250 μL insert prior to LC-MS/MS analysis. Quantification of PFAS was performed using a TSQ Altis Plus triple quadrupole mass spectrometer (Thermo Fisher Scientific) coupled with a Vanquish ultra-performance liquid chromatography (UPLC) system (Thermo Fisher Scientific). A 5.0 μL aliquot of the sample extract was injected, and chromatographic separation was carried out at 30 °C on an ACQUITY UPLC BEH C18 column (1.7 μm, 2.1 × 50 mm; Waters, cat#186002350) equipped with a VanGuard BEH C18 pre-column (1.7 μm, 2.1 × 5 mm; Waters, cat#186003975). An isolator column (2.1 × 50 mm; Waters, cat#186004476) was installed between the mixer and the injection valve to act as a PFAS delay column. The mobile phases consisted of (A) 5 mM ammonium acetate in LC-MS water (Thermo Fisher Scientific) and (B) methanol. The UPLC gradient was performed at a flow rate of 0.2 mL/min as follows: 5% B for 1.0 min, increased to 60% B over 1.0 min, ramped to 100% B over 6.0 min, held at 100% B for 2.0 min, returned to 5% B over 0.1 min, and held at 5% B for 4.8 min for column re-equilibration. The first 3 min of UPLC elutes were diverted to waste to remove salts and potential coextracted polar components. Mass spectrometric detection was conducted using negative electrospray ionization (ESI) at a spray voltage of −2.8 kV in selected reaction monitoring (SRM) mode. The optimized SRM transitions and other MS parameters are summarized in Table S2. The ion transfer tube temperature, vaporizer temperature, sheath gas flow, and auxiliary gas flow were set to 325 °C, 350 °C, 50 AU and 10 AU, respectively.

PFOA was present in a linear form in the dosing solutions, calibration standards, and samples, whereas PFOS consisted of both linear and branched isomers. All quantification was performed on the sum of linear and branched PFOS, consistent with the calibration standard (100% linear PFOA and 78.8% linear PFOS). As a result, we are unable to provide comments on the toxicology of branched versus linear structures. All samples below the detection range were redefined as half of the detection limit.

#### Biochemistry

Collected blood was left to clot for 15–30 min at room temperature and was centrifuged at 2,000 x g. The supernatant (serum) was transferred to a new tube. Serum alkaline phosphatase (ALP), alanine aminotransferase (ALT), calcium (Ca^2+^), cholesterol (CHOL), free thyroxine (FT4), lactate dehydrogenase (LDH), total bilirubin (TBI), and triglycerides (TGL) were measured on the Siemens Dimension EXL 200 Integrated Chemistry System with the Dimension software (Table S3).

To evaluate the relationship between nominal versus measured PFAS exposure benchmark doses (BMDs) were derived for highly responsive serum biomarkers (ALP and ALT) using the *tcplfit2* package in R (Sheffield et al., 2022). Concentration–response modeling was performed separately for PFOA and PFOS using both nominal doses (daily administered dose multiplied by the study duration of 56 days to obtain cumulative mg/kg) and measured liver concentrations (average concentration within each dosing group). Vehicle control responses were used to define the baseline; for measured liver concentrations, vehicle controls were assigned a nominal concentration of zero to anchor baseline responses. BMD modeling was conducted using the *concRespCore* function, and the model producing the best fit was used to estimate the BMD, BMDL, and BMDU.

BMD estimates were filtered according to four criteria: (1) hit probability ≥0.9, (2) BMDs within the experimental dose range, (3) finite BMDL and BMDU values, and (4) a BMDU/BMDL ratio <40. BMDs failing any of these filters were excluded from downstream analyses. Filtered BMDs were visualized using log-scaled dot–whisker plots to compare nominal and measured dose–response relationships across PFAS and biomarkers.

#### Liver samples RNA isolation and purification

Snap-frozen livers in liquid nitrogen were cut on dry ice using a cooled scalpel, as adopted in previous studies employing similar methods.^76^^,^^77^ Tissue resection after snap-freezing was adopted to allow timely tissue processing at necropsy and preserve RNA integrity.^78^ 12–25 mg of liver were homogenized in PowerBead tubes (Qiagen, cat#13114-50) containing 2.8 mm ceramic beads and 600 μL of RLT buffer from the RNeasy Mini Kit (Qiagen, cat#74104) supplemented with 6 μL of β-mercaptoethanol (Millipore Sigma, cat#M3148-25 ML). Homogenization was carried out in a Qiagen PowerLyzer 24 Homogenizer (two 45 s cycles at 3500 rpm with a 30 s pause in between). Following homogenization, the lysate was transferred to a new tube and centrifuged at 13,000 x g for 2 min. The supernatant (lysate) was transferred to a new tube, and ribonucleic acid (RNA) was extracted with Qiagen RNeasy Mini Kit according to the manufacturer’s instructions (HB-0435-006, Version 10/2019). 600 μL of 70% ethanol (Commercial Alcohols, cat#P016EA95) was added to the lysate, mixed by inversion, transferred to the RNeasy spin column (two rounds of up to 700 μL lysate transferred), spun (flow-through discarded). All centrifugations were performed at 8,000 x g for 15 s at room temperature, unless stated otherwise. DNA digestion was performed using the RNase-free DNase Set (Qiagen, cat#79254). Before and after DNA digestion, 350 μL of RW1 buffer was added to the RNeasy spin column, spun and flow through (wash). Digestion was done with 80 μL of diluted DNase I (1:7 ratio; DNase: RDD buffer) incubated on the RNeasy spin column membrane for 15 min at room temperature. Following DNA digestion, the RNeasy was washed twice with 500 μL of RPE buffer. The optional centrifugation (full speed for 1 min at room temperature) of the RNeasy spin column in a new 2 mL collection tube was performed. DNA was eluted in 40 μL of RNase-free water (spun at 8,000 x g for 1 min at room temperature). A second elution was done by reapplying the 40 μL of RNase-free water from the first elution onto the RNeasy spin column. RNA concentration, purity, and integrity were assessed with the Thermo Fisher Scientific Nanodrop ND-1000 spectrophotometer (ND 1000 3.7 software) and the Agilent TapeStation 4200 system (Agilent 2200 TapeStation Controller software). The TapeStation RNA assessment was done using the RNA sample buffer (Agilent, cat#5067–5577) and the RNA ScreenTape (Agilent, cat#5067–5576) according to the manufacturer’s instructions (G2964-90022, Revision D). In short, 1 μL of RNA was mixed with 5 μL of RNA sample buffer and briefly spun to bring down all liquid. Samples were denatured for 3 min at 72°C, put on ice for 2 min and briefly spun again before being loaded into the Agilent TapeStation 4200 system. Reagents were brought up to room temperature before use. Note that an electronic ladder with the Agilent 2200 TapeStation Controller software was used for the analysis. RNA Integrity Number (RIN) ranged from 6.4 to 8.7, with 98% of samples above or equal to 7.0 (average: 7.9 ± 0.5; median: 7.9), indicating sample suitability for sequencing.

#### TempO-seq sequencing library preparation

The TempO-Seq mouse S1500+ surrogate gene panel (version 1.2; BioSpyder Technologies Inc., cat#KT-06-096) was used to generate sequencing libraries for measuring gene expression in total RNA purified from mouse liver tissues. Each RNA sample was first diluted to 100 ng/mL in water, then mixed with an equal volume of 2× TempO-Seq Lysis buffer, resulting in a final RNA concentration of 50 ng/μL. From this, 2 μL of this diluted mixture (approximately 100 ng of RNA) was used as input for TempO-Seq library building. As per the manufacturer’s protocol (Protocol version: Document 100802 rev C), RNA was first hybridized with the detection oligos (DOs). Unbound DOs were digested with a nuclease, and the remaining bound DOs were ligated. These ligated oligos were then amplified by PCR using primers with sequencing tags to create the sequencing libraries. The resulting libraries were pooled together and quantified using quantitative PCR (qPCR) with the KAPA SYBR FAST Universal kit (Roche, cat#07960140001), which is compatible with Illumina platforms. The pooled libraries were sequenced using a 75-cycle flow cell on an Illumina NextSeq 500.

#### Sequencing data quality control and processing

BCL files were converted to FASTQ format and demultiplexed using bcl2fastq (v2.20). Processing and quality control of the FASTQ files, differential expression analysis, and exploratory statistical analyses were performed using the Omics Data Analysis Frameworks for Regulatory application.^62^ The pipeline utilizes Snakemake (v8.0)^63^ and scripts written in R (v.4.3.2) to manage workflows. FASTA and GTF-formatted reference files were produced using the manifest provided by BioSpyder. First, fastp (v0.23.2) was used for trimming,^64^ followed by STAR (v2.7.8a)^65^ to align raw reads to the reference sequence. Feature counts were extracted from the aligned reads (BAM files) using the qCount function of the QuasR R package (v1.30.0),^66^ according to the features specified in a GTF file. A samples-by-probes count matrix was generated. Across experimental samples, the median number of mapped reads was 1,148,263.

Quality control was performed on the count matrices to identify and remove outliers and low-quality samples, using the methods in Harrill et al. (2021) as a guideline. A Spearman’s correlation coefficient cutoff of 0.1 was applied within each group to remove samples that were poorly correlated with others in the same treatment group. As per Harrill et al. (2021), the cut-off for uniquely mapped reads was set at 10% of the number of target sequences (e.g., for a target of 1,000,000 reads, at least 100,000 reads must pass the filter for the sample to be retained). Any samples outside of Tukey’s Outer Fence (3x interquartile range) for the following criteria were removed: the count of probes accounting for the top 80% of the signal, and the number of detected probes with at least five mapped reads. Samples with a Gini coefficient, an indicator of inequality in distributions, greater than 0.95 were excluded.^68^

The count matrices that passed quality control were imported into R for statistical analysis. Following the R-ODAF guidelines,^62^ probes were filtered to retain only those for which at least 75% of the samples in an experimental group had counts above 0.5 counts per million (CPM). Additionally, spurious spikes were eliminated by excluding probes where the difference between the maximum and median counts was less than the total counts divided by the number of replicates plus one.

Using DESeq2 version 1.38.0,^67^ differentially expressed genes (DEGs) were identified in count matrices that passed filters by comparing dose groups to their respective vehicle controls within each sex. Log_2_ fold change shrinkage was performed using the ashr method.^79^ Results were extracted at an alpha threshold of 0.05 and reported as Wald test *p*-values, with false discovery rate (FDR) adjustment for multiple testing. DEGs were filtered using a linear fold change cutoff of 1.5 and an adjusted *p*-value threshold of 0.05 for downstream analyses.

The TempO-seq count data are available on NCBI’s Gene Expression Omnibus (GEO) under the accession number GSE303480.

#### Benchmark dose modeling

BMDExpress3 (https://github.com/auerbachs/BMDExpress-3) was used to model dose-response curves of individual genes for each chemical within each sex. Log_2_-transformed and normalized gene expression data were used as input.^80^ First, the Williams Trend Test was conducted on genes with a >1.5-fold linear change, using 500 permutations to filter out non-responsive genes with a *p*-value >0.01. BMD for a benchmark response of 1 standard deviation (SD) above vehicle control was calculated for each gene using the EPA BMDS,^68^^,^^69^ which identifies the best-fitting model among Power, Exp3, Exp5, Linear, and Poly2 models and selects the BMD calculated using that model. Constant variance and the profile likelihood BMDU (upper limit) and BMDL (lower limit) estimation method were selected as parameters. BMD values were then filtered to remove genes with BMDs that were higher than the highest tested dose, those with a BMDU/BMDL ratio ≥40, and/or a best-fit *p*-value of ≤0.05.

The resulting BMD list for each chemical within each sex was used for pathway analyses within BMDExpress3 to calculate BMDs of signaling pathways and biological processes associated with the dose-responsive genes. Furthermore, the list of dose-responsive genes at day 56 and the DEGs at day 28 for each chemical were used in KEGG analysis to identify additional potential pathways affected by the exposures. To enable comparison between sexes and PFAS type, q-values were grouped by sex or by PFAS compound and summarized using the minimum q-value across all relevant exposure conditions (PFOA vs. PFOS, female vs. male, 28- and 56-day exposures).

#### T cell dependent antibody response assay

For the following immunotoxicity sections, data acquisition was performed only on the 56-day cohort. Based on OECD Guidelines^81^ five days before euthanasia, T cell-dependent antibody response (TDAR) was elicited in vehicle, PFOA, and PFOS-gavaged groups through the administration of 100 μL of 25% Sheep Red Blood Cells (SRBC) suspension by intraperitoneal injection. 10% SRBC solution (Rockland, cat#R405-0050) was washed three times with PBS (centrifugation at 1,000 xg, 10 min at 4°C). The supernatant was removed between each wash and SRBC were resuspended to obtain a 25% solution after the third wash. SRBCs are T cell-dependent antigens that can initiate antibody production.

At euthanasia, blood was collected, and plasma was isolated. Plasma anti-SRBCs immunoglobulin M (IgM) levels at euthanasia were assessed using a mouse anti-SRBCs IgM enzyme-linked immunosorbent assay (ELISA; Life Diagnostics, cat#SRBCM-1) according to manufacturer instructions (Revision 02222022), the recommended plasma dilution (50-fold) was utilized. In brief, 100 μL of samples and standards, and blank (diluent) were added per well (in duplicate). Plates were incubated on a micro-plate shaker at 150 rpm for 45 min at room temperature. All incubations were done with the same agitation and temperature. The plates were sealed before each incubation. Plates were washed with wash solution five times manually using a squeeze bottle and inverted sharply on absorbent paper to remove residual wash buffer. 100 μL of enzyme conjugate was added, incubated for 45 min, and washed five times. 100 μL of TMB reagent was added, incubated protected from light for 25 min. Reaction was stopped with 100 μL of stop solution and briefly mixed before data acquisition at 450 nm (within 5 min). Data was acquired using the Biotek Synergy H4 Hybrid reader with Gen 5.3.10 software. Samples below the assay’s detection range were redefined as half of the standard curve’s lowest concentration (detection limit).

#### Cytokine measurements

Plasma was isolated according to the Bio-Plex Pro Cytokine, Chemokine, and Growth Factor Assays instruction manual (Bio-Rad, Manual#10000142118, Version A). In brief, blood collected by cardiac puncture was incubated in K2 EDTA blood collection tubes (BD, cat#365974) at room temperature for 30–60 min. Blood was centrifuged twice (1,000 xg for 15 min and 10,000 xg for 10 min; both at 4°C) and the supernatant was collected and stored at −80°C after the second centrifugation.

Cytokine concentrations at euthanasia were assessed using the Bio-Plex Mouse Cytokine Group 1 Panel 23-Plex (Bio-Rad, cat#M60-009RDPD) according to the kit’s instructions (Manual# 10014905, Version A). In brief, plasma was diluted three or 4-fold based on volume availability. 50 μL of coupled beads were added per well and washed twice. Washes were performed using the Bio-Plex Pro II Wash Station (Bio-Rad; MAGx2 or MAGx3 programs). 50 μL of standards, blank, and samples were added per well in duplicate, incubated for 30 min, and washed three times. All incubations were done at 850 rpm at room temperature, protected from light. 25 μL of detection was added per well, incubated for 30 min, and washed three times. 50 μL of streptavidin-PE was added per well, incubated for 10 min, and washed three times. 125 μL of assay buffer was added per well. Plates were mixed for 30 s at 850 rpm (protected from light) before data acquisition. Data was acquired on the Bio-Plex 200 System (Bio-Rad) using the Bio-Plex Manager 6.1 Software (Bio-Rad). The Bio-Plex 200 System was validated and calibrated before data acquisition with the Bio-Plex validation (Bio-Rad, cat#171–203001) and calibration (Bio-Rad, cat#171–203060) kits, respectively. Again, samples below the assay’s detection range were defined as one-half of the limit of detection.

#### Flow cytometry

Flow cytometry was performed on whole blood, spleen, and thymus. Blood collected by cardiac puncture was incubated in K2 EDTA blood collection tubes (BD, cat#365974) at room temperature for 30–60 min. Spleen halves and thymuses were collected in PBS and stored on ice until dissociation. Spleen dissociation was performed with Miltenyi gentleMACS Octo Dissociator with Heaters (Miltenyi, cat#130-096-427) and Miltenyi mouse spleen dissociation kit (Miltenyi, cat#130-095-926) according to the manufacturer’s instructions (protocol version 140-003-084.05). In brief, spleen halves were added to a GentleMACS C tube containing 2.4 mL of Buffer S, 50 μL of Enzyme D, and 15 μL of Enzyme A. Spleens were dissociated using GentleMACS program 37C_m_SDK_1. Following the digestion, the spleens were sieved through a 70 μm strainer (Thermo Fisher Scientific, cat#22-363-548) and the filter was rinsed with 2.5 mL of Buffer S. The thymuses were transferred onto a 70 μm cell strainers (Thermo Fisher Scientific, cat#22-363-548) placed over a 50 mL tubes. Using the rubber end of a 3 mL syringe plunger (BD, cat#309657), the thymuses were mechanically dissociated (gentle pressure and circular motion to release single cells). The filter was rinsed with 2.5 mL of PBS. Spleen and thymus filtrates were centrifuged (580xg for 8 min at 4°C) and resuspended in 1 mL of PBS. Approximately, 1 x 10^5^ to 1 x 10^7^ spleen and thymus cells were transferred to round-bottom tubes, and volume was completed to 100 μL with PBS.

Viability staining and fragment crystallizable region (Fc) blocking were performed with the BD fixable viability stain 510 (BD, cat#564406) and BD purified rat anti-mouse CD16/CD32 (BD, cat#553142), respectively, according to the manufacturer’s instructions (protocol 564406 revision 1 and protocol 553142 revision 17). In brief, 10 μL of 10-fold diluted viability stain was added to the single cell suspension and incubated for 15 min at 4°C protected from light. All incubations were done at 4°C protected from light, unless stated otherwise. Cells were washed once with 2 mL of stain buffer (BD, cat#554656) and resuspended in 100 μL of stain buffer. All centrifugations before fixation were performed at 440 xg for 5 min. 10 μL of 10-fold diluted Fc block was added to the cells and incubated for 5 min. Extracellular staining (Table S4) was done for 30 min at 4°C. Note that brilliant stain buffer plus (BD, cat#566385) was used during the extracellular staining (10μL/sample). Red blood cell lysis (Invitrogen, cat#00-4300-54) was performed on blood and spleen samples following the manufacturer’s instructions (revision 10). Briefly, 2 mL of 1X red blood cell lysis buffer was added to the samples, pulse vortexed to mix, and incubated 5–10 min at room temperature. Samples were washed twice with 2 mL of stain buffer. Fixation was performed using the fixative from the BD transcription factor buffer set (BD, cat#562574) according to the manufacturer’s instructions (protocol 562574 revision 6). In short, cells were fixed with 1 mL of 1X Fix/Perm (adding while mixing) for 40 min. Cells were washed once with 1 mL of cold PBS, resuspended in 500 μL of cold PBS, and stored overnight at 4°C protected from light. All centrifugations after fixation were performed at 380 xg for 6 min at 4°C. The next morning, cells are washed twice with 2 mL of 1X Perm/Wash buffer. Cells were centrifuged and resuspended in 100 μL (blood) or 350 μL (spleen and thymus) of stain buffer before acquisition.

Data was acquired on a BD LSRFortessa Cell Analyzer (five lasers; ultraviolet/violet/blue/yellow-green/red), where 15–25,000 viable CD3^+^/CD4^+^ events were acquired (based on fixable viability stain 510). Gates were set using fluorescence minus one (FMO) control. Compensation beads (Invitrogen cat#01-2222-42 and cat#A10346) were used following the manufacturer’s instructions (publication MAN0019374, revision C, and publication MAN0002029 (MP10346), revision B.0) as single-color controls. Briefly, one drop of compensation beads (Invitrogen, cat#01-2222-42) was incubated for 30 min at 4°C protected from light with one antibody (same volumes as extracellular staining; Table S4). The compensation controls were introduced in the sample protocol stated above at the red blood cell lysis step. For the viability stain single-color control, one drop of reactive beads (Invitrogen, cat#A10346) was mixed with 0.5 μL of viability stain (10X) and incubated for 30 min at room temperature protected from light. Beads were washed with 3 mL of PBS and spun at 400 xg for 5 min. Beads were resuspended in 500 μL of staining buffer, and one drop of negative beads was added. The analysis was performed using FlowJo (v10.9.0).

### Quantification and statistical analysis

Figures and statistical analyses were performed in R (v4.3.2).^82^ Data is presented as heat maps (median) or as boxplots (median ±1.5 IQR with individual values). Statistical significance was assessed using the Kruskal-Wallis test followed by Dunn’s multiple comparisons test due to a lack of normality among one or more groups and small sample size, where the vehicle group was defined as the “control”. Statistical significance was defined as follows: ∗*p* ≤ 0.05, ∗∗*p* ≤ 0.01, ∗∗∗*p* ≤ 0.001, and ∗∗∗∗*p* ≤ 0.0001. Select *p*-values between 0.099 and 0.05 are also presented (#). Code available at https://github.com/HC-EHSRB-CompTox. Statistical details of each experiment can be found in the figure legends.
