# Supplementary material for: PFOS and PFOA exposure induces liver injury and sex-dependent immune effects in C57BL/6 mice
Source: iScience. 2026 Jan 14;29(2):114693. doi: 10.1016/j.isci.2026.114693 (PMC12865548; doi:10.1016/j.isci.2026.114693)
Supplement: Document S1. Figures S1–S12 and Tables S1–S4 [file mmc1.pdf]

iScience, Volume *29*

Supplemental information

PFOS and PFOA exposure induces liver injury and sex-dependent immune effects in C57BL/6 mice

Amélie Blais, Allison Loan, Eunnara Cho, Asia Woodtke, Houman Moteshareie, Lauren M. Bradford, Gong Zhang, Guillaume Pelletier, Martha Navarro, Matthew J. Meier, Andy Nong, Rocio Aranda-Rodriguez, Kristin M. Eccles, David Prescott, and Azam F. Tayabali

Supplementary Figures

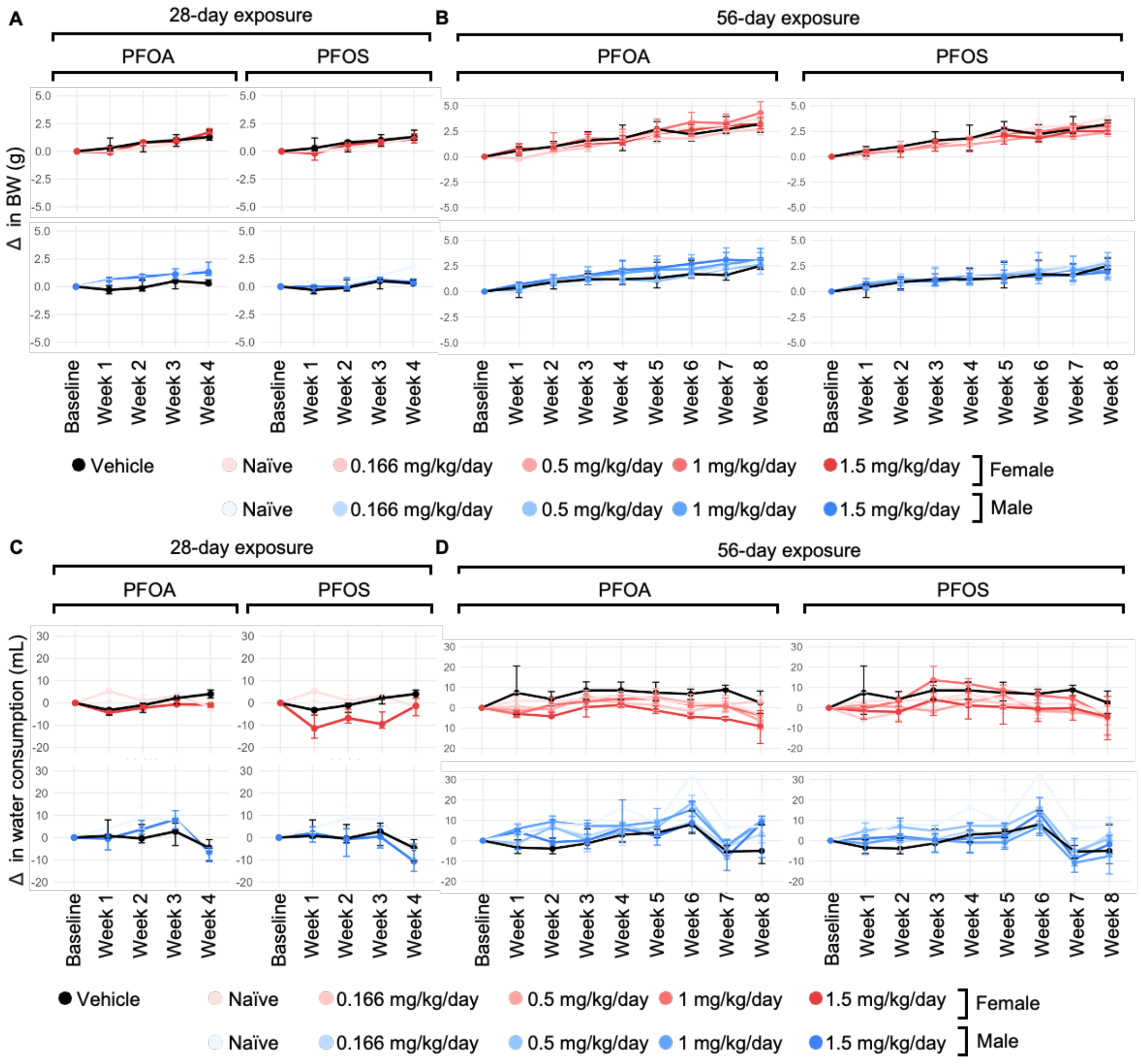

Figure S1. Weekly body weight and water consumption. (A-B) Change in body weight (g) of PFOA or PFOS-exposed male and female mice after 28 (A) or 56 (B) days of treatment relative to day 1 (“delta” baseline). Change from baseline was calculated by subtracting each value from the corresponding baseline value within each sample. n=8 mice/group. (C-D) Water consumption of PFOA and PFOS-exposed male and female mice after 28 (C) or 56 (D) days of treatment relative to day 1 (baseline). Female water consumption (mL) per mouse was obtained by dividing the cage water consumption by the number of mice per cage. N = 7-8 mice/group (2-4 cages/group for females). Statistical significance was assessed using the Kruskal-Wallis test with Dunn’s multiple comparisons (control: vehicle) for each time point. Data is presented as medians ± 1.5 IQR. Abbreviations: BW, body weight; IQR, interquartile range; PFOA, perfluorooctanoic acid; PFOS, perfluorooctanesulfonic.

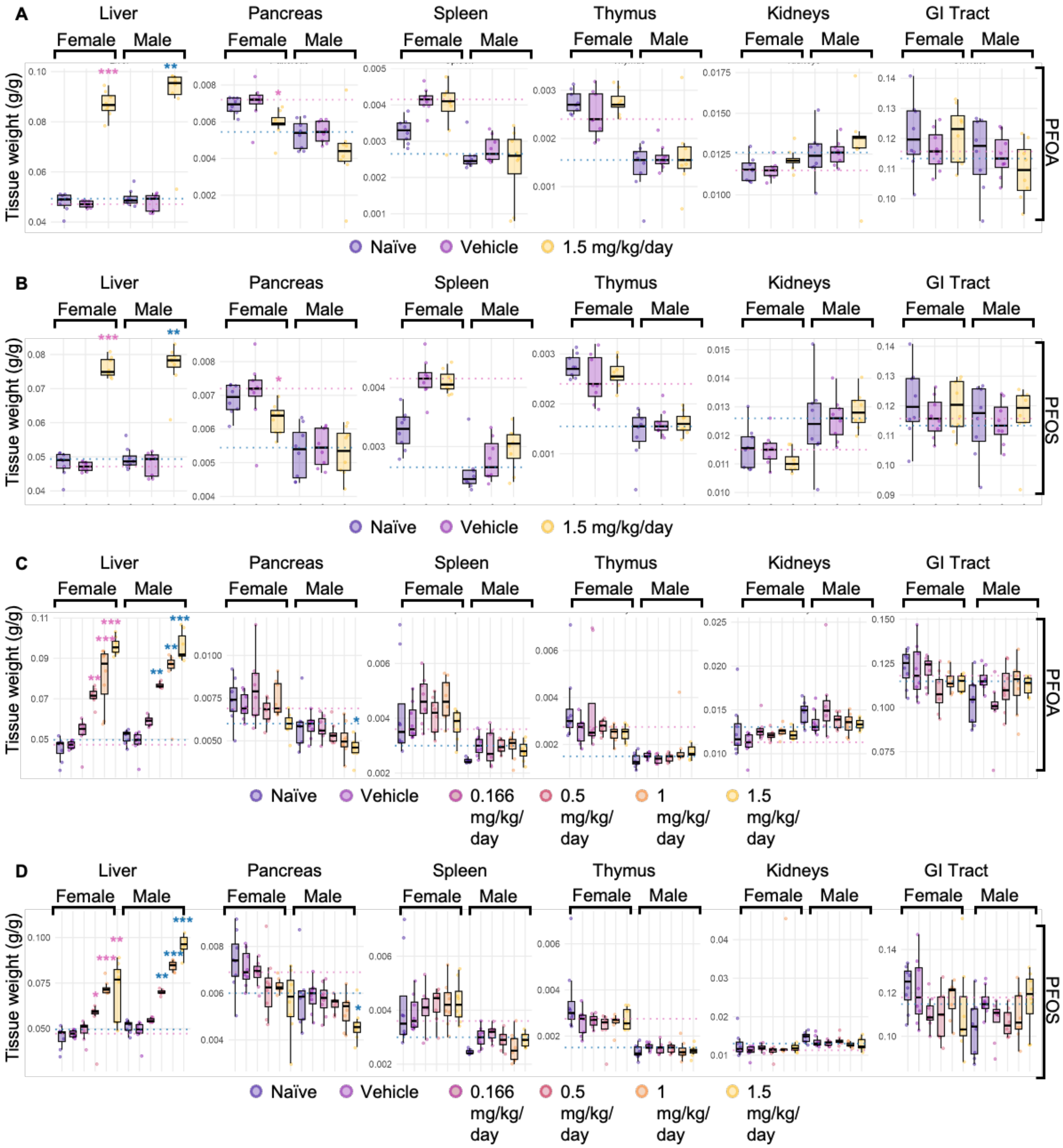

Figure S2. PFOA and PFOS exposure results in hepatomegaly and pancreatic atrophy. (A-D) Box plots of liver, pancreas, spleen, thymus, kidneys, and GI tract weight normalized to euthanasia body weight in PFOA (A,C) and PFOS (B,D) exposed mice following 28 (A-B) or 56 (C-D) days of exposure. Data is presented as box plots ± 1.5 IQR and median (line). Individual biological replicates are shown as points. n=8 mice/group. Statistical significance was assessed using the Kruskal-Wallis test with Dunn's multiple comparisons (control: vehicle) for each parameter. *$p \leq 0.05$; **$p \leq 0.01$; ***$p \leq 0.001$. Abbreviations: GI, gastrointestinal tract; IQR, interquartile range; PFOA, perfluorooctanoic acid; PFOS, perfluorooctanesulfonic acid.

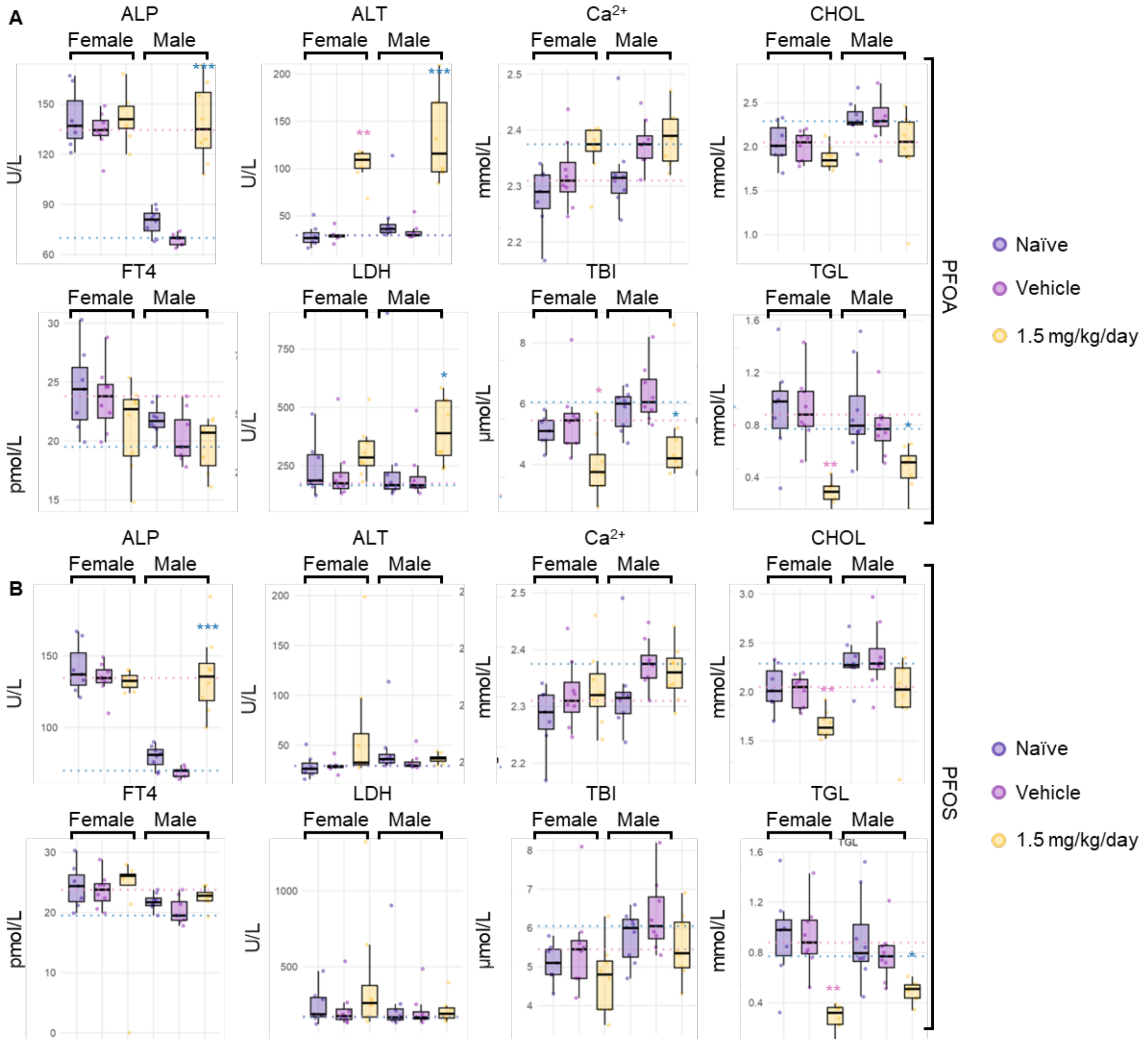

Figure S3. PFOA and PFOS induced liver toxicity following 28 days of exposure. (A-B) Box plots of serum ALP, ALT, Ca^2, CHOL, FT4, LDH, TBI, and TGL at euthanasia in PFOA (A) and PFOS (B) exposed mice following 28 days of exposure. Data is presented as box plots ± 1.5 IQR and median (line). Individual biological replicates are shown as points. n=8 mice/group. Statistical significance was assessed using the Kruskal-Wallis test with Dunn's multiple comparisons (control: vehicle) for each parameter. *p≤0.05; **p≤0.01; ***p≤0.001. Abbreviations: ALP, alkaline phosphatase; ALT, alanine aminotransferase; Ca^2, calcium; CHOL, cholesterol; FT4, free thyroxine; IQR, interquartile range; LDH, lactate dehydrogenase; PFOA, perfluorooctanoic acid; PFOS, perfluorooctanesulfonic acid; TBI, total bilirubin; TGL, triglycerides.

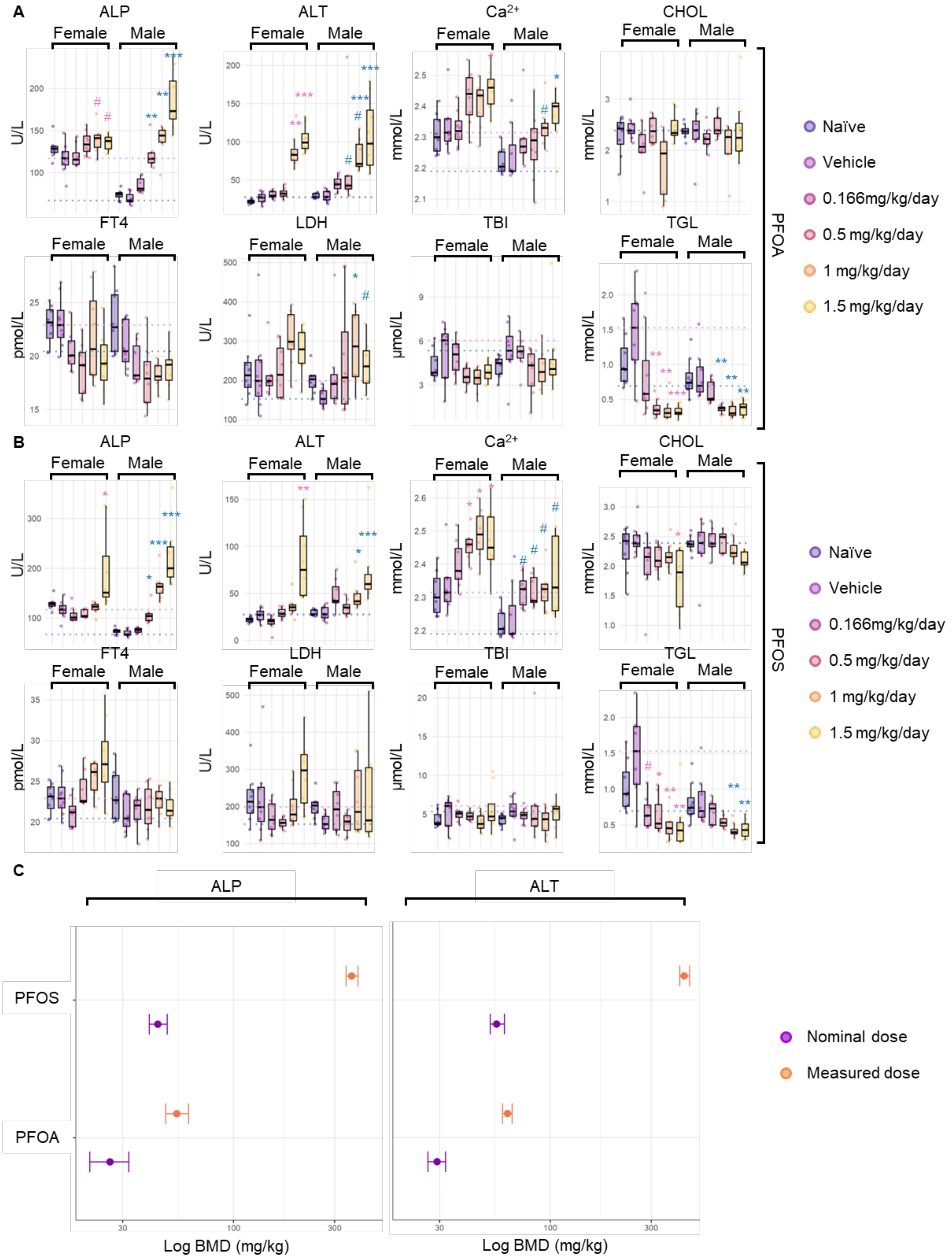
A
ALP
ALT
Ca2+
CHOL
FT4
LDH
TBI
TGL
Female
Male
U/L
mmol/L
pmol/L
µmol/L
PFOA
B
PFOS
Naïve
Vehicle
0.166mg/kg/day
0.5 mg/kg/day
1 mg/kg/day
1.5 mg/kg/day
C
PFOS
PFOA
Log BMD (mg/kg)
Nominal dose
Measured dose

Figure S4. PFOA and PFOS induced liver toxicity following 56 days of exposure. (A-B) Box plots of serum ALP, ALT, Ca^2, CHOL, FT4, LDH, TBI, and TGL at euthanasia in PFOA (A) and PFOS (B) exposed mice following 56 days of exposure. Data is presented as box plots ± 1.5 IQR and median (line). Individual biological replicates are shown as points. n=8 mice/group. Statistical significance was assessed using the Kruskal-Wallis test with Dunn's multiple comparisons (control: vehicle) for each parameter. #p≤0.099; *p≤0.05; **p≤0.01; ***p≤0.001. (C) Comparison of BMDs for highly responsive serum biomarkers (ALP and ALT) following PFOA and PFOS exposure. BMDs were derived using the *tcplfit2* package from either nominal cumulative doses (mg/kg, calculated as daily dose × 56 days) or measured liver concentrations. Error bars indicate BMD lower and upper confidence limits. n=8 mice/group. Abbreviations: ALP, alkaline phosphatase; ALT, alanine aminotransferase; BMD, benchmark dose; Ca^2, calcium; CHOL, cholesterol; FT4, free thyroxine; IQR, interquartile range; LDH, lactate dehydrogenase; PFOA, perfluorooctanoic acid; PFOS, perfluorooctanesulfonic acid; TBI, total bilirubin; TGL, triglycerides.

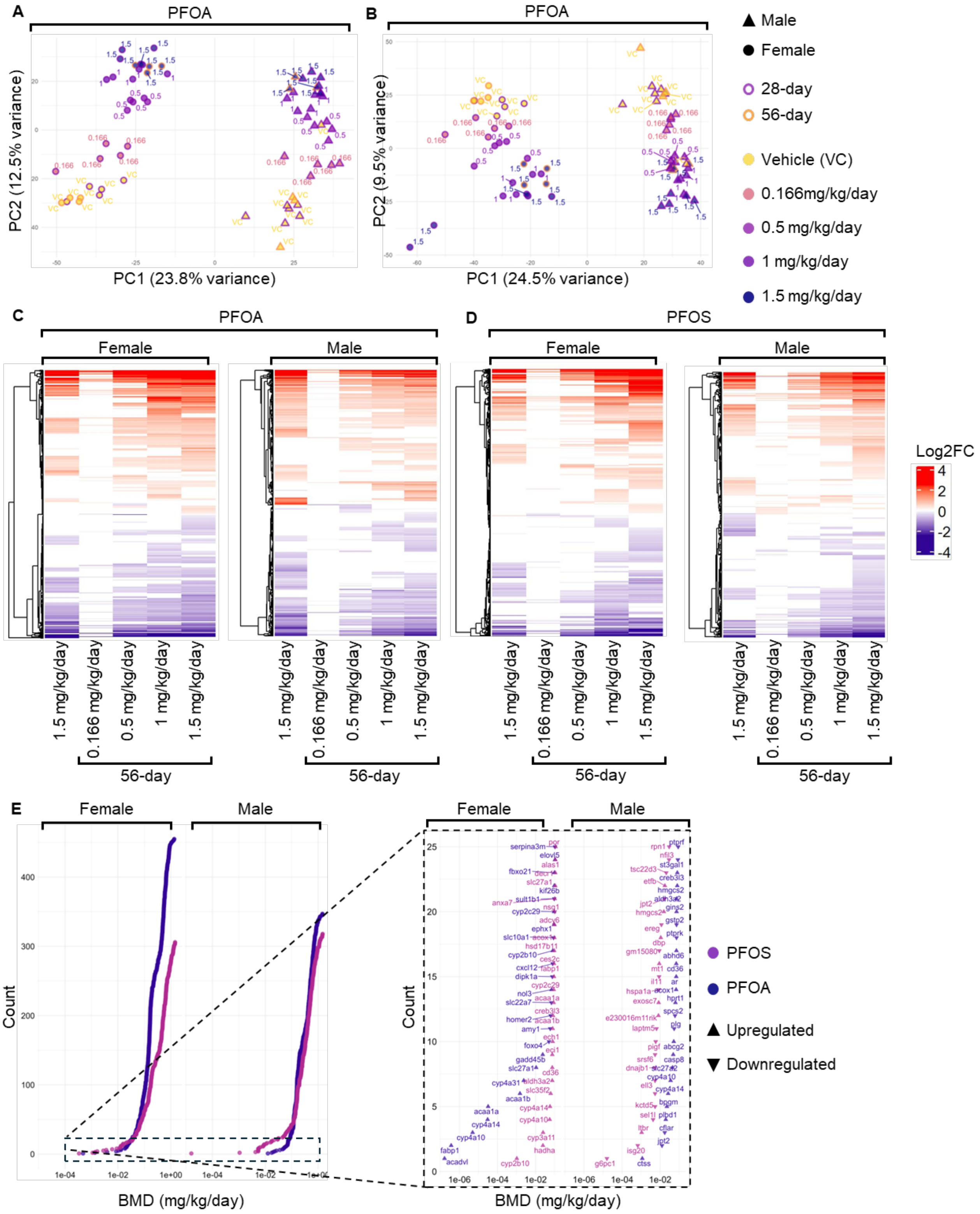

Figure S5. Transcriptomic analysis of PFAS-exposed mouse livers. (A-B) PCA of PFOA (A) and PFOS (B) treatment groups. Colored by dosage group, shaped by sex, and outlined by time point. (C-D) Heatmaps of DEGs in PFOA (C) and PFOS (D) treatment groups. DEGs were defined as those with FC > 1.5 and $p_{adj} < 0.05$. (E) BMD analysis of DEGs in the 56-day treatment cohort. Genes with a BMDU/BMDL ratio ≥ 40 and/or best-fit model p-value

≤ 0.05 were considered responsive. Abbreviations: DEG, differentially expressed gene; Log 2FC, Log2 fold change; p_{adj}, adjusted p-value; PFAS, per- and polyfluoroalkyl substances; PFOA, perfluorooctanoic acid; PFOS, perfluorooctanesulfonic acid; PCA, principal component analysis.

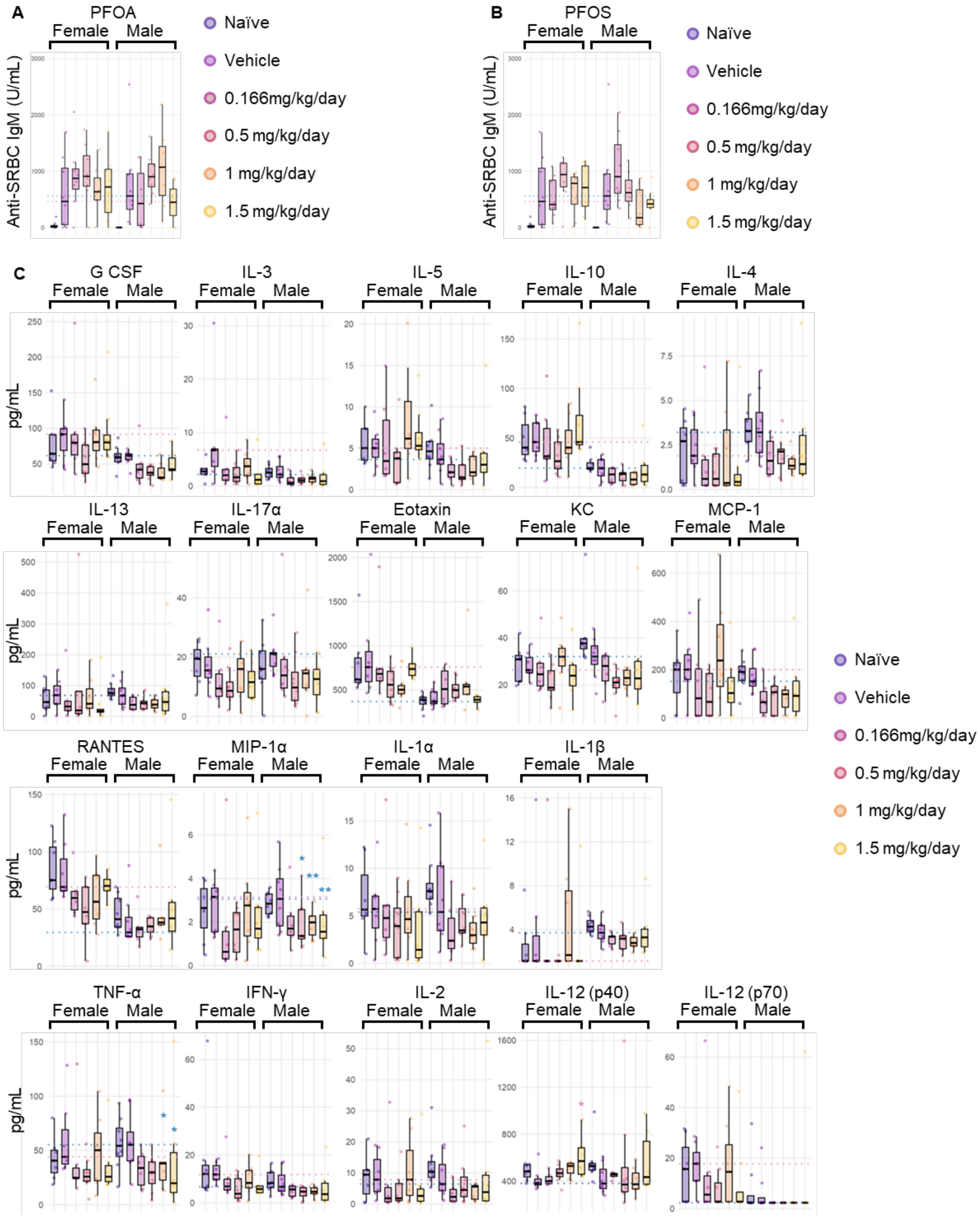

Figure S6. T Cell Dependent Antibody Response and PFOA-induced immune dysregulation marked by plasma cytokines. (A-B) Plasma anti-SRBC IgM concentration at euthanasia in PFOA (A) and PFOS (B) exposed mice. n=8 mice/group. (C) Box plots of 19 cytokines in PFOA-exposed mice following 56 days of exposure. Data is presented as box plots ± 1.5 IQR and median (line). Individual biological replicates are shown as points. n=8 mice/group.

Statistical significance was assessed using the Kruskal-Wallis test with Dunn's multiple comparisons (control: vehicle) for each parameter. #$p \leq 0.099$; *$p \leq 0.05$; **$p \leq 0.01$; ***$p \leq 0.001$. Abbreviations: CCL5 (RANTES), C-C motif chemokine ligand 5; G-CSF, granulocyte colony-stimulating factor; IFN-γ, interferon-gamma; IgM, immunoglobulin M; IL, interleukin; IQR, interquartile range; KC, keratinocyte chemoattractant; MCP-1, monocyte chemoattractant protein-1; MIP, macrophage inflammatory protein; PFOA, perfluorooctanoic acid; PFOS, perfluorooctanesulfonic acid; SRBC, sheep red blood cells; TNF-α, tumor necrosis factor-alpha.

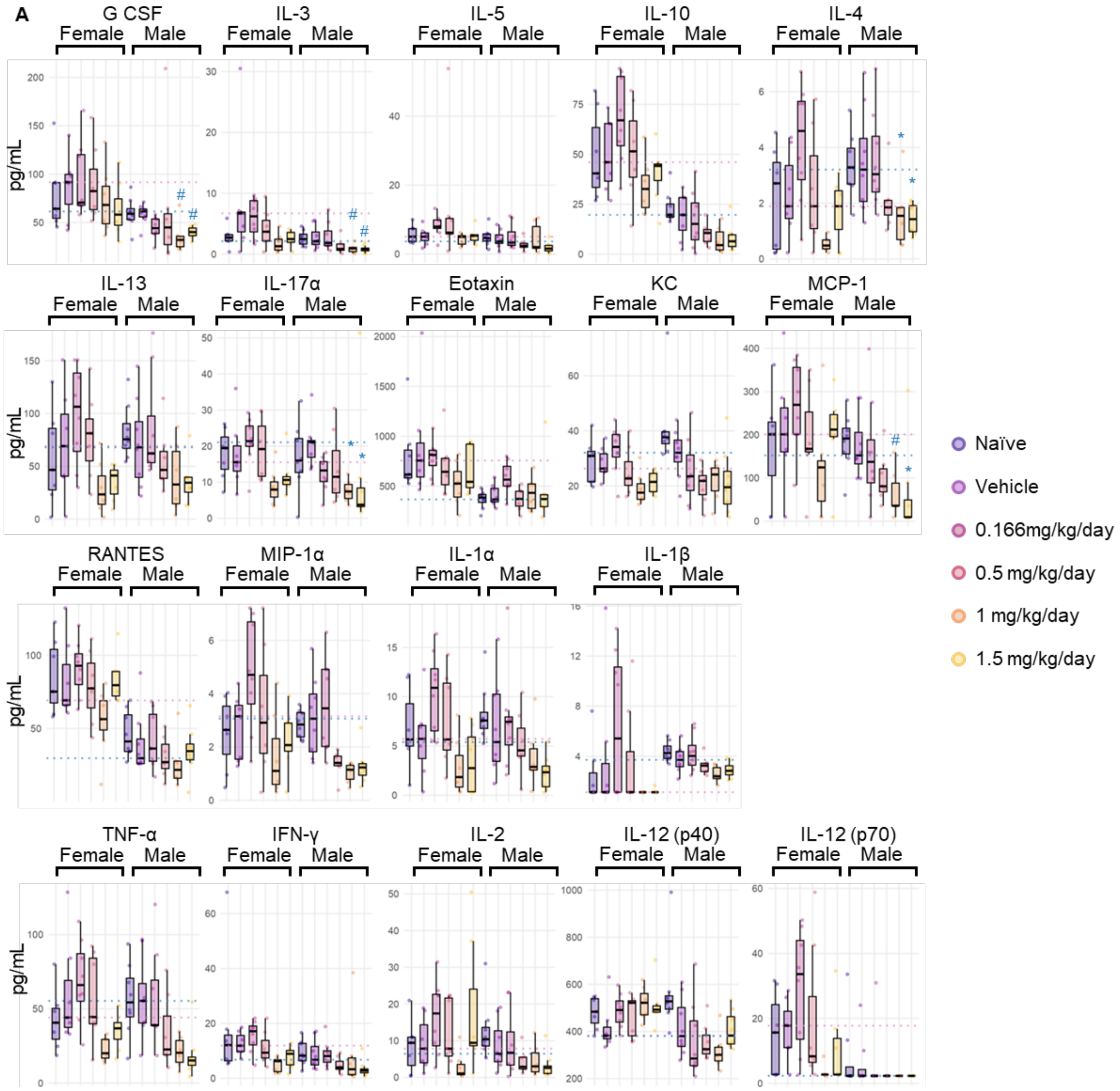

Figure S7. PFOS-induced immune dysregulation marked by plasma cytokines. (A) Box plots of 19 cytokines in PFOS-exposed mice following 56 days of exposure. Data is presented as box plots ± 1.5 IQR and median (line). Individual biological replicates are shown as points. n=8 mice/group. Statistical significance was assessed using the Kruskal-Wallis test with Dunn's multiple comparisons (control: vehicle) for each parameter. #p≤0.099; *p≤0.05; **p≤0.01; ***p≤0.001. Abbreviations: G-CSF, granulocyte colony-stimulating factor; IFN-γ, interferon-gamma; IL, interleukin; IQR, interquartile range; KC, keratinocyte chemoattractant; MCP-1, monocyte chemoattractant protein-1; CCL5 (RANTES), C-C motif chemokine ligand 5; MIP, macrophage inflammatory protein; PFOS, perfluorooctanesulfonic acid; PFOA, perfluorooctanoic acid; TNF-α, tumor necrosis factor-alpha.

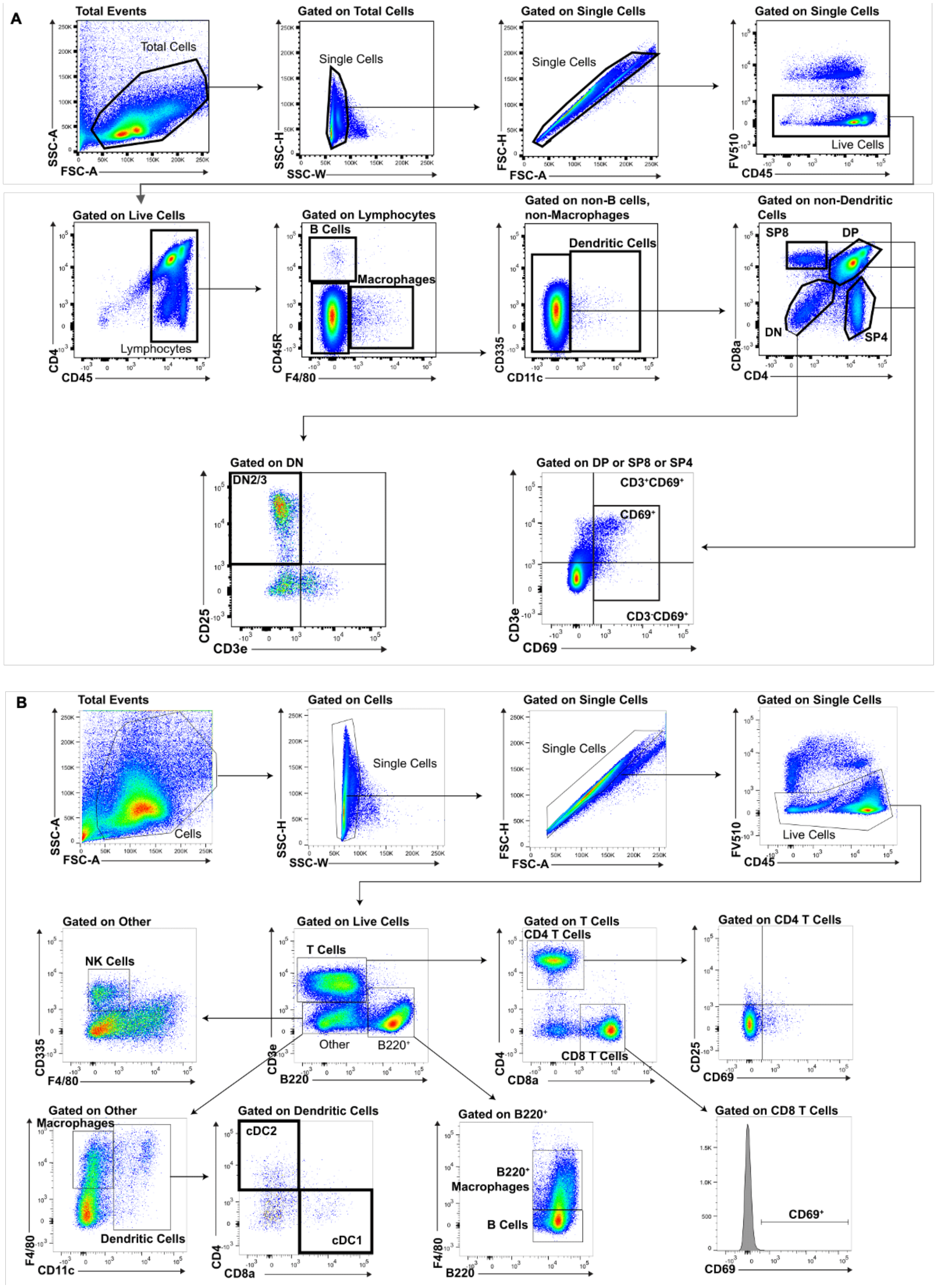
A
Total Events
Total Cells
SSC-A
FSC-A
Gated on Total Cells
Single Cells
SSC-H
SSC-W
Gated on Single Cells
Single Cells
FSC-H
FSC-A
Gated on Single Cells
Live Cells
FV510
CD45
Gated on Live Cells
Lymphocytes
CD4
CD45
Gated on Lymphocytes
B Cells
Macrophages
CD45R
F4/80
Gated on non-B cells, non-Macrophages
Dendritic Cells
CD335
CD11c
Gated on non-Dendritic Cells
SP8
DP
DN
SP4
CD8a
CD4
Gated on DN
DN2/3
CD25
CD3e
Gated on DP or SP8 or SP4
CD3⁺CD69⁺
CD69⁺
CD3⁻CD69⁺
CD3e
CD69
B
Total Events
Cells
SSC-A
FSC-A
Gated on Cells
Single Cells
SSC-H
SSC-W
Gated on Single Cells
Single Cells
FSC-H
FSC-A
Gated on Single Cells
Live Cells
FV510
CD45
Gated on Other
NK Cells
CD335
F4/80
Gated on Live Cells
T Cells
Other
B220⁺
CD3e
B220
Gated on T Cells
CD4 T Cells
CD8 T Cells
CD4
CD8a
Gated on CD4 T Cells
CD25
CD69
Gated on Other
Macrophages
Dendritic Cells
F4/80
CD11c
Gated on Dendritic Cells
cDC2
cDC1
CD4
CD8a
Gated on B220⁺
B220⁺ Macrophages
B Cells
F4/80
B220
Gated on CD8 T Cells
CD69⁺
CD69

Figure S8. (A) Flow cytometry gating strategy in the thymus and the (B) spleen.

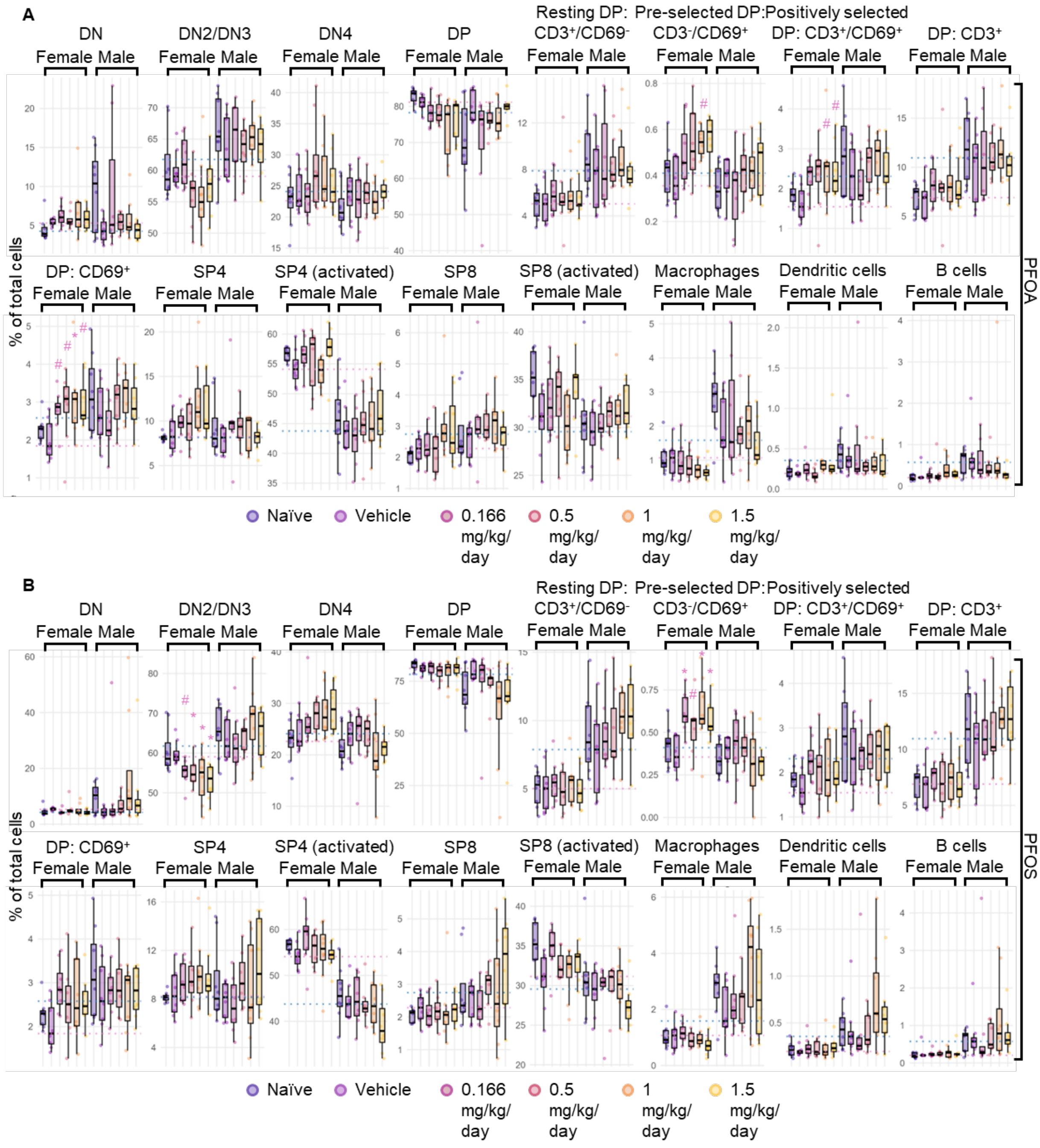

Figure S9. PFOA and PFOS cause changes in thymocyte development in female mice. (A-B) Box plots of thymus cell populations at euthanasia following exposure to PFOA (A) or PFOS (B). Data is presented as box plots ± 1.5 IQR and median (line). Individual biological replicates are shown as points. n=7-8 mice/group. Statistical significance was assessed using the Kruskal-Wallis test with Dunn's multiple comparisons (control: vehicle) for each parameter. #$p \leq 0.099$; *$p \leq 0.05$; **$p \leq 0.01$; ***$p \leq 0.001$. Abbreviations: DN, double negative ($CD4^-/CD8^-$) thymocytes; DN2/DN3, double negative stage 2/3 ($CD4^-/CD8^-/CD25^+$) thymocytes; DN4, double negative stage 4 ($CD4^-/CD8^-/CD25^-$) thymocytes; DP, double positive ($CD4^+/CD8^+$) thymocytes; IQR, interquartile range; PFOS, perfluorooctanesulfonic acid; PFOA, perfluorooctanoic acid; SP4, single positive ($CD4^+/CD8^-$) T cells; SP8, single positive ($CD4^-/CD8^+$) T cells.

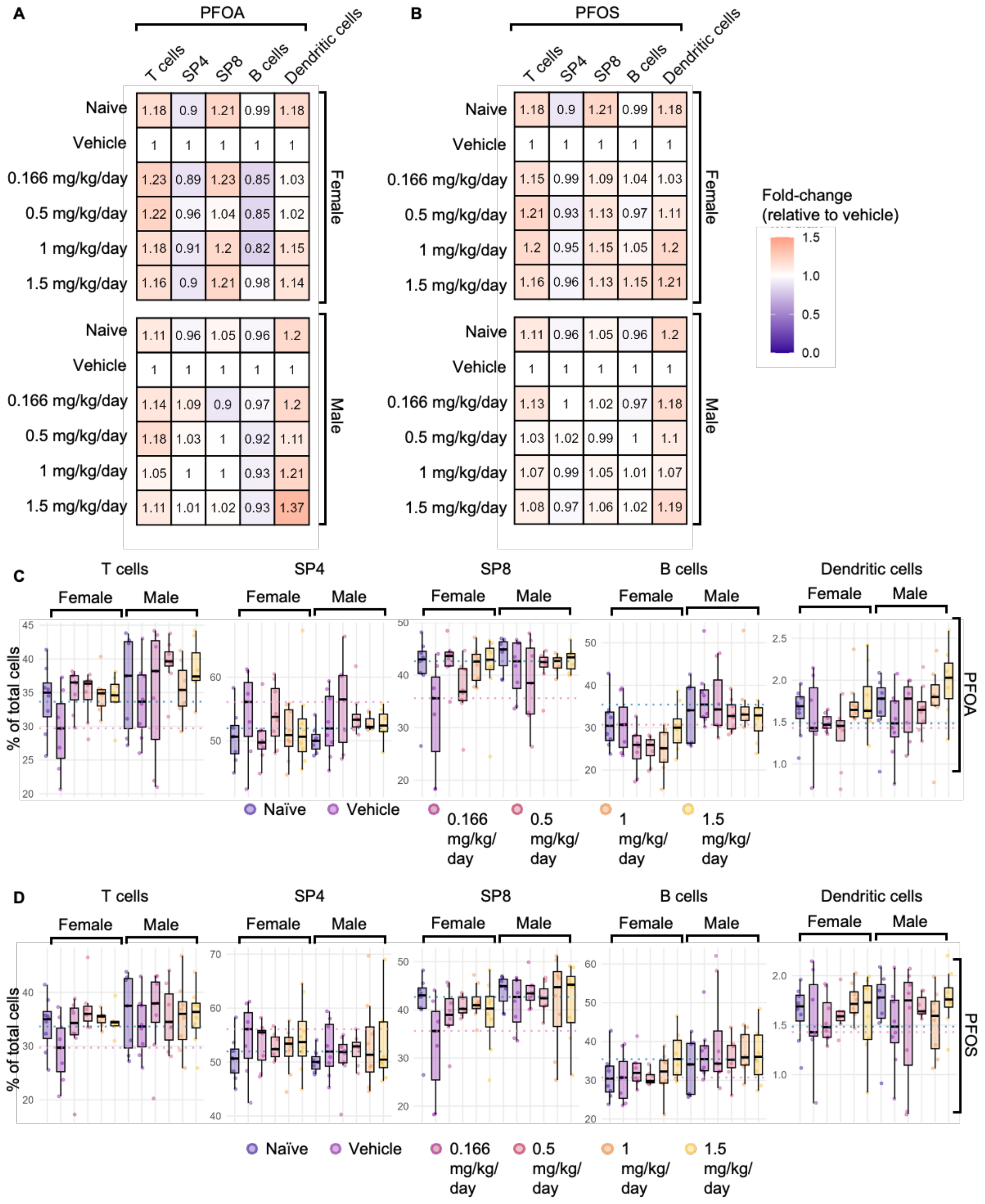

Figure S10. PFOA and PFOS do not cause changes in splenic populations. (A-B) Median fold change (vs same-sex vehicle) of splenic cell populations at euthanasia following exposure to PFOA (A) or PFOS (B). Data is presented as median fold change. (C-D) Box plots of splenic cell populations at euthanasia following exposure to PFOA (C) or PFOS (D). Data is presented as box plots ± 1.5 IQR and median (line). Individual biological replicates are shown as

points. n=7-8 mice/group. Abbreviations: IQR, interquartile range; PFOS, perfluorooctanesulfonic acid; PFOA, perfluorooctanoic acid; SP4, single positive ($CD4^+/CD8^-$) T cells; SP8, single positive ($CD4^-/CD8^+$) T cells.

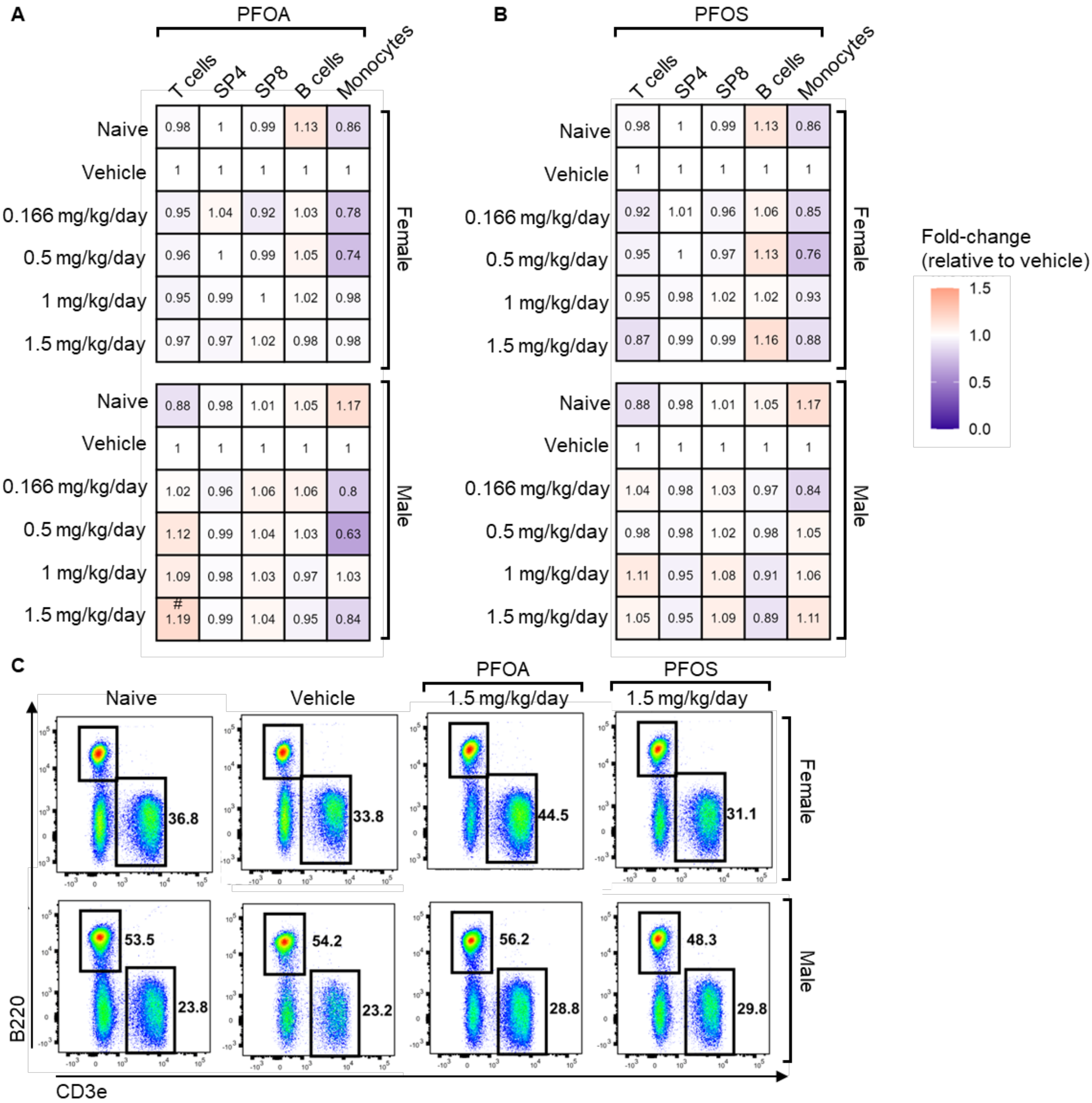

Figure S11. PFOA and PFOS cause changes in the T cell population in the blood. (A-B) Median fold change (vs same-sex vehicle) of cell populations in the blood at euthanasia following exposure to PFOA (A) or PFOS (B). n=6-8. Statistical significance was assessed using the Kruskal-Wallis test with Dunn's multiple comparisons (control: vehicle) for each parameter. Data is presented as median fold change. (C) Representative dot plots for B220/CD3e staining in naïve, vehicle, 1.5 mg/kg/day PFOA, and 1.5 mg/kg/day PFOS-treated males and females. #$p \leq 0.099$. Abbreviations: PFOS, perfluorooctanesulfonic acid; PFOA, perfluorooctanoic acid; SP4, single positive ($CD4^+/CD8^-$) T cells; SP8, single positive ($CD4^-/CD8^+$) T cells.

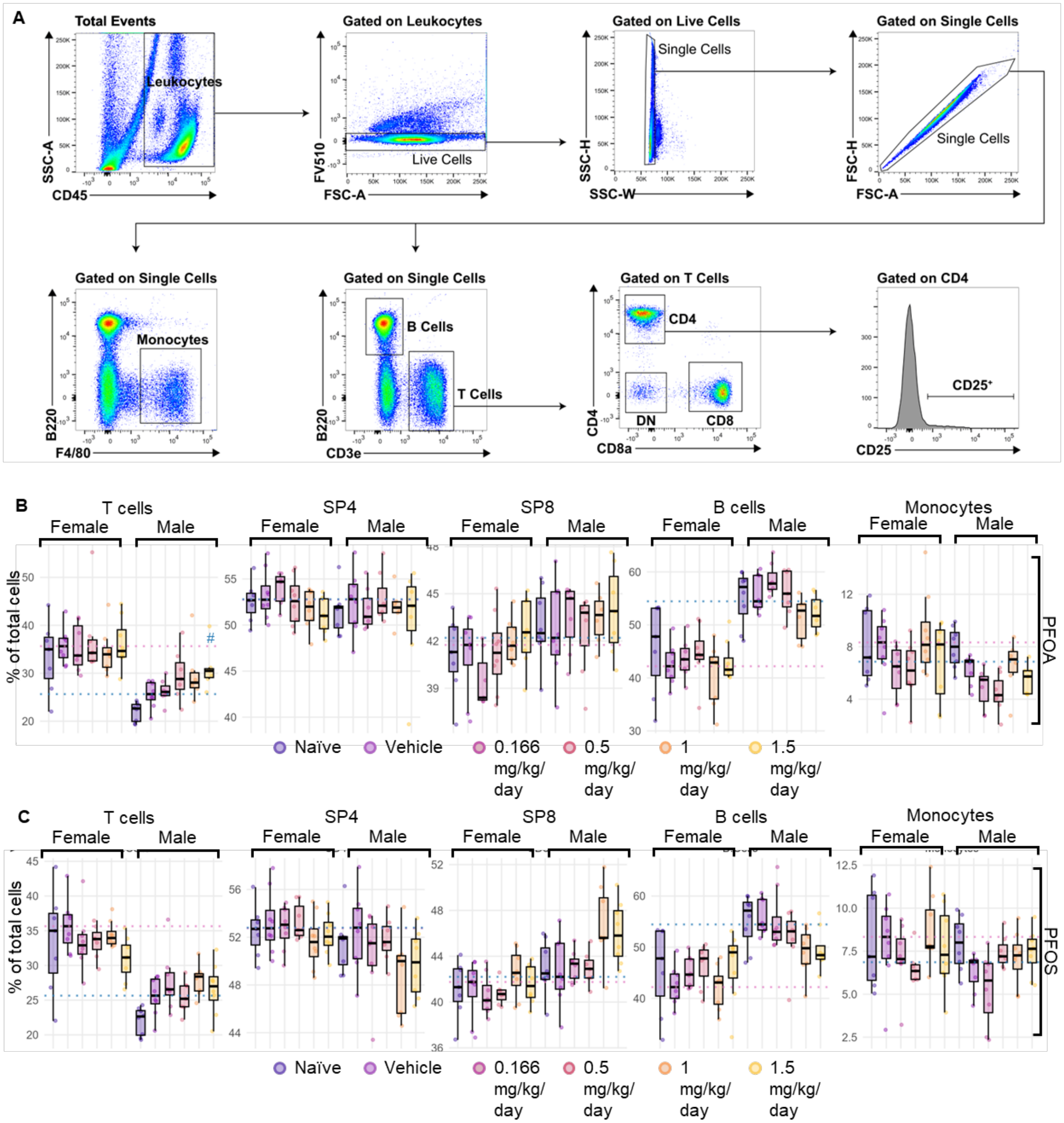

Figure S12. PFOA and PFOS cause changes in the T cell population in the blood. (A) Flow cytometry gating strategy in the blood. (B-C) Box plots of cell populations in the blood at euthanasia following exposure to PFOA (B) or PFOS (C). Data is presented as box plots ± 1.5 IQR and median (line). Individual biological replicates are shown as points. n=6-8 mice/group. Statistical significance was assessed using the Kruskal-Wallis test with Dunn's multiple comparisons (control: vehicle) for each parameter. #p≤0.099. Abbreviations: PFOS, perfluorooctanesulfonic acid; PFOA, perfluorooctanoic acid; SP4, single positive ($CD4^+/CD8^-$) T cells; SP8, single positive ($CD4^-/CD8^+$) T cells.

Supplementary Tables

Table S1. Environmental Enrichment and Bedding.

Enrichment Type	Product (Manufacturer, cat#)
**Bedding***	Performance Bedding (Biofresh, cat#L0107)
**Nesting material***	Alpha Twist (Shepherd Specialty Papers, cat#AT06118)
**Gnawing device***	Bed-r'Nest (White Paper Pucks; The Andersons, cat#BRN8WSR)
	Aspen Wood Stick (Lomir Biomedical, cat#AC-S01)
**Shelter, tunnel, and exercise device**	Mouse Tunnel (Bio Serv, cat#K3322/K3323/K3487)**
	Fast-Trac (Bio Serv, cat#K3250/K3251)*
	Mouse Igloo (Bio Serv, cat#K3327/K2217/K3570)**
**Treat**	Sunflower Seeds (Bio Serv, cat#S5137)
	Rodent Foraging crumbles, Bacon flavoured (Bio Serv, cat#S5783)***
**Supplement*****	HydroGel (Clear H_2O, cat#70-01-5022)***
	Nutra-Gel Diet (Bio Serv, cat#S5769-TRAY)***

* Replaced weekly.
** Replaced every 2 weeks.
*** Given to certain mice when signs of dehydration or stress arose.

Table S2. Compound name, acronym, CAS number, and key mass spectrometer parameters. Targeted PFAS and internal standard (IS), including RF lens voltage, selective reaction monitoring (SRM) transitions, collision energy (CE), and instrument detection limits (IDL).

Compounds Name	CAS	Acronym	RF (V)	Quantifier		Qualifier		IDL ng/mL
				Transition	CE (V)	Transition	CE (V)	
**Perfluorobutanoic acid**	375-22-4	PFBA	31	213.0>168.9	8.6	NA	NA	0.008
**Perfluoropentanoic acid**	2706-90-3	PFPeA	32	263.0>218.9	7.8	NA	NA	0.009
**Perfluorohexanoic acid**	307-24-4	PFHxA	37	313.9>268.9	8.3	NA	NA	0.011
**Perfluoroheptanoic acid**	375-85-9	PFHpA	43	363.0>318.9	9.1	363.0>168.9	17.2	0.005
**Perfluorooctanoic acid**	335-67-1	PFOA	47	413.0>368.9	9.3	413.0>168.9	17.8	0.016
**Perfluorononanoic acid**	375-95-1	PFNA	51	463.0>418.9	9.6	463.0>218.9	16.3	0.015
**Perfluorodecanoic acid**	335-76-2	PFDA	56	513.0>468.9	10.4	513.0>218.9	17.9	0.021
**Perfluoroundecanoic acid**	2058-94-8	PFUdA	59	563.0>518.9	10.7	563.0>268.9	18.6	0.007
**Perfluorododecanoic acid**	307-55-1	PFDoA	65	612.9>568.9	11.6	612.9>168.9	27.0	0.011
**Perfluorotridecanoic acid**	72629-94-8	PFTrDA	73	662.9>618.9	12.1	662.9>168.9	28.2	0.010
**Perfluorotetradecanoic acid**	376-06-7	PFTeDA	71	712.9>668.9	12.3	NA	NA	0.011
**Perfluorobutane sulfonic acid**	375-73-5	PFBS	112	298.8>79.8	35.0	298.8>98.8	30.6	0.015
**Perfluoropentane sulfonic acid**	2706-91-4	PFPeS	126	348.8>79.8	40.3	348.8>98.8	34.7	0.025
**Perfluorohexane sulfonic acid**	355-46-4	PFHxS**	111	398.8>79.8	42.8	398.8>98.8	37.9	0.016
**Perfluoroheptane sulfonic acid**	375-92-8	PFHpS	161	448.8>79.8	46.1	448.8>98.8	40.8	0.019
**Perfluorooctane sulfonic acid**	1763-23-1	PFOS**	120	498.8>79.8	51.6	498.8>98.8	44.6	0.023
**Perfluorononane sulfonic acid**	68259-12-1	PFNS	250	548.8>79.8	57.1	548.8>98.8	48.4	0.016
**Perfluorodecane sulfonic acid**	335-77-3	PFDS	119	598.8>79.8	57.7	598.8>98.8	50.8	0.026
**4:2 Fluorotelomer sulfonate**	757124-72-4	4:2-FTS	101	327.0>306.9	19.7	327.0>79.8	35.7	0.007
**6:2 Fluorotelomer sulfonate**	27619-97-2	6:2-FTS	124	427.0>406.8	23.1	427.0>80.8	34.9	0.028

**8:2 Fluorotelomer sulfonate**	39108-34-4	8:2-FTS	120	527.0>506.8	26.8	527.0>486.8	33.0	0.027
***N-Methylperfluorooctane sulfonamidoacetic acid**	2355-31-9	N-MeFOSAA	96	569.9>511.9	22.5	569.9>418.9	20.3	0.029
***N-Ethylperfluorooctane sulfonamidoacetic acid**	2991-50-6	N-EtFOSAA	89	583.9>525.9	21.0	569.9>418.9	20.3	0.017
***Perfluorooctane sulfonamide**	754-91-6	FOSA	119	497.9>477.8	24.5	497.9>77.8	36.3	0.010
**Internal standards**								
**Perfluoro-n-(13C) butanoic acid**	NA	MPFBA	31	217.0>172.0	8.6	NA	NA	NA
**Perfluoro-n-(13C) pentanoic acid**	NA	M5PFPeA	32	268.0>223.0	7.8	NA	NA	NA
**Perfluoro-n-(1,2,3,4,6-13C) hexanoic acid**	NA	M5PFHxA	37	318.0>273.0	8.3	NA	NA	NA
**Perfluoro-n-(1,2,3,4-13C) heptanoic acid**	NA	M4PFHpA	45	366.9>322.0	9.1	NA	NA	NA
**Perfluoro-n-(13C) octanoic acid**	NA	M8PFOA	47	421.0>376.0	9.3	NA	NA	NA
**Perfluoro-n-(13C) nonanoic acid**	NA	M9PFNA	51	472.0>427.0	9.6	NA	NA	NA
**Perfluoro-n-(1,2,3,4,5,6-13C) decanoic acid**	NA	M6PFDA	56	519.0>474.0	10.4	NA	NA	NA
**Perfluoro-n-(1,2,3,4,5,6,7-13C) undecanoic acid**	NA	M7PFUdA	59	570.0>525.0	10.7	NA	NA	NA
**Perfluoro-n-(1,2-13C) dodecanoic acid**	NA	MPFDoA	67	614.9>569.9	11.5	NA	NA	NA
**Perfluoro-n-(1,2-13C) tetradecanoic acid**	NA	M2PFTeDA	71	715.0>670.0	12.3	NA	NA	NA
**Sodiumperfluoro-1-(2,3,4-13C) butanesulfonate**	NA	M3PFBS	113	302.0>80.0	31.6	NA	NA	NA
**Sodiumperfluoro-1-(1,2,3-13C) hexanesulfonate**	NA	M3PFHxS	111	402.0>99.0	37.9	NA	NA	NA
**Sodiumperfluoro-1-(13C) octanesulfonate**	NA	M8PFOS	121	507.0>99.0	45.6	NA	NA	NA
**Sodium1H,1H,2H,2H-perfluoro(1,2-13C) hexanesulfonate**	NA	M2-4:2 FTS	101	329.0>309.0	19.7	NA	NA	NA

**Sodium1H,1H,2H,2H-perfluoro(1,2-13C) octanesulfonate**	NA	M2-6:2 FTS	124	429.0>409.0	23.1	NA	NA	NA
**Sodium1H,1H,2H,2H-perfluoro(1,2-13C) decanesulfonate**	NA	M2-8:2 FTS	120	529.0>509.0	26.8	NA	NA	NA
**N-methyl-d-perfluoro-1-octanesulfonamidoacetic acid**	NA	d3-N-MeFOSAA	92	572.9>418.9	20.3	NA	NA	NA
**N-ethyl-d-perfluoro-1-octanesulfonamidoacetic acid**	NA	d5-N-EtFOSAA	93	589.0>418.9	20.8	NA	NA	NA
**Perfluoro-1-(13C) octanesulfonamide**	NA	M8FOSA	119	506.0>78.0	24.5	NA	NA	NA

IDL is estimated based on the quantifier transition.
* N-MeFOSAA, N-EtFOSSA and FOSA were not included in the μSPE method but included in the dilute-and-shoot method.
** PFHxS and PFOS are mixtures that contain linear and a variety of branched isomers.

Table S3. Serum Biochemistry Reagents.

Category	Product (Manufacturer, cat#)
**General Reagents**	System Check Reagent Cartridge (Siemens, 10481507)
	Purified Water Diluent (Siemens, 10444887)
	Chemistry Wash (Siemens, 10445052)
	Sample Diluent (Siemens, 10444875)
	Reagent Probe Cleaner (Siemens, 10445036)
	Sample Probe Cleaner (Siemens, 10445035)
	Alkaline Phosphatase Reagent Cartridge (Siemens, 10642445)
	Alanine Aminotransferase Reagent Cartridge (Siemens, 10475530)
	Calcium Reagent Cartridge (Siemens, 10444949)
	Cholesterol Reagent Cartridge (Siemens, 10444891)
	Lactate Dehydrogenase Reagent Cartridge (Siemens, 10284483)
	Free thyroxine (FT4L) Reagent Cartridge (Siemens, 10464524)
	Total Bilirubin Reagent Cartridge (Siemens, 10444957)
	Triglycerides Reagent Cartridge (Siemens, 10444906)
**Calibration**	Chemistry I Multiple Analytes Calibrator (Siemens, 10716280)
	Chemistry II Multiple Analytes Calibrator (Siemens, 10444997)
	Enzyme I Calibrator (Siemens, 10284680)
	Enzyme II Calibrator (Siemens, 10476170)
	Alkaline Phosphatase Calibrator (Siemens, 10714028)
	Cholesterol Calibrator (Siemens, 10444998)
	Total Bilirubin/Direct Bilirubin Calibrator (Siemens, 10445013)
	Thyroid Multiple Analytes Calibrator (Siemens, 10484354)
**Consumables**	Cuvette Cartridge (Siemens, 10445042)
	Sample Cup (Siemens, 10445041)

Table S4. Flow Cytometry Antibodies. Flow cytometry antibodies and buffers used for extracellular staining, with their volume per one million cells.

Antibody/Buffer	Volume ($\mu L/10^6$ cells)
**PE Hamster Anti-Mouse CD69 (BD, 561932)**	0.30
**FITC Hamster Anti-Mouse CD3e (BD, 553061)**	0.50
**PE-CF594 Rat Anti-Mouse F4/80 (BD, 565613)**	0.60
**BV421 Hamster Anti-Mouse CD11c (BD, 562782)**	1.25
**PE-Cy ™ 7 Rat Anti-Mouse CD25 (BD, 552880)**	1.25
**BV786 Rat Anti-Mouse CD45R/B220 (BD, 563894)**	1.25
**BUV395 Rat Anti-Mouse CD4 (BD, 563790)**	2.50
**R718 Rat Anti-Mouse CD8a (BD, 566985)**	2.50
**PerCP-Cy ™ 5.5 Rat Anti-Mouse CD45 (BD, 550994)**	2.50
**BUV737 Rat Anti-Mouse CD335 (NKp46) (BD, 612805)**	5.00
**Brilliant Stain Buffer Plus (BD, 566385)**	10.00 *
**Stain Buffer (FBS) (BD, 554656)**	22.35 **

* Volume per test
** Complete volume to 50 μL
